# Dietary fats suppress the peritoneal seeding of colorectal cancer cells through the TLR4/Cxcl10 axis in adipose tissue macrophages

**DOI:** 10.1038/s41392-020-00327-z

**Published:** 2020-10-15

**Authors:** Wei Xiang, Rongchen Shi, Dapeng Zhang, Xia Kang, Lili Zhang, Jing Yuan, Xuan Zhang, Hongming Miao

**Affiliations:** 1 Department of Biochemistry and Molecular Biology, Third Military Medical University (Army Medical University), 400038 Chongqing, China; 2 Department of Oncology, Southwest Hospital, Third Military Medical University (Army Medical University), 400038 Chongqing, China

**Keywords:** Tumour immunology, Cancer microenvironment, Cancer therapy

## Abstract

Peritoneal carcinomatosis (PC) of colorectal cancer (CRC) is a terminal phase of malignancy with no effective strategies for the prevention of this condition. Here we established PC models in mice by intraperitoneal engraftment of CRC cells and revealed an unexpected role for a high-fat diet (HFD) in preventing metastatic seeding in the visceral fat. Mechanistically, the HFD stimulated the activation of adipose tissue macrophages (ATMs) toward an M1-like phenotype and enhanced ATM tumor phagocytosis in a TLR4-dependent manner. Furthermore, the TLR4–Cxcl10 axis in ATMs promoted T cell recruitment, and M1-like macrophages stimulated T cell activation in tumor-seeded fats. The inhibitory effect of the HFD on tumor seeding was abolished with the ablation of macrophages, inactivation of T cells, or blockade of the TLR4–Cxcl10 axis in macrophages. Finally, we showed that a HFD and conventional chemotherapeutic agents (oxaliplatin or 5-fluorouracil) synergistically improved the survival of tumor-seeded mice. Collectively, our findings demonstrate that peritoneal seeding of CRC can be suppressed by short-term treatment with a HFD in the early phase, providing a novel concept for the management of these patients in the clinic.

## Introduction

Colorectal cancer (CRC), with a major cause of related mortality being metastasis, is the third most common type of malignancy.^1^ In addition to the hematogenous and lymphatic routes, peritoneal carcinomatosis (PC) often occurs in CRC. CRC-PC is encountered in approximately 7% of patients at primary surgery, up to 19% of patients during follow-up after curative surgery, and up to 80% of patients who succumb to CRC.^2^ Patients with CRC-PC are generally considered to be in the terminal phase and receive only supportive treatment, including palliative surgery and chemotherapy.^3^ 5-Fluorouracil (5FU), oxaliplatin (OXP), leucovorin, and irinotecan are commonly used chemotherapeutic agents for CRC-PC treatment that are administered in a single or combined manner and achieve a median survival time not exceeding 12.6 months, according to previous reports.^2,4,5^ Therefore, exploration of preventive strategies for CRC-PC is urgently needed.

PC generally goes through three major stages including peritoneal dissemination from primary tumors, seeding in the microenvironment of targeted tissues, and fast growth in the terminal phase. Peritoneal dissemination from primary CRC frequently results from surgery-related rupture of the bowel and vascular system or primary tumors breaking through the bowel wall.^6,7^ However, it is still unclear how disseminated CRC cells seed and grow in target tissues.

In patients, CRC-PC mainly targets the omentum, a large fat pad that extends from the stomach and covers the bowel. This phenomenon is similar to the peritoneal metastasis of serous ovarian cancer, in which 80% of cases undergo omental metastases.^8,9^ To study PC in mouse models, intraperitoneal engraftment of cancer cells (CRC cells,^10,11^ ovarian cancer,^12,13^ etc.) is commonly used. Nieman et al. established a PC model of ovarian cancer by intraperitoneal injection of ovarian cancer cells into nude mice and found that adipocytes could provide energy for the rapid growth of metastatic cancer cells.^12^ We established PC models with CRC cells or melanoma cells in previous studies and found that PC progression was intensively regulated by the microenvironment of the visceral fat.^10,11,14^

Fat tissues are infiltrated by a variety of immune cells, among which macrophages play central roles in regulating local and systemic inflammation and the immune status.^15^ A high-fat diet (HFD), which contains high levels of saturated fatty acids, can convert adipose tissue macrophages (ATMs) from the M2-like phenotype (CD11c^−^CD206^+^) to the M1-like (CD11c^+^CD206^−^) phenotype and induce chronic inflammation in fat tissues and other organs.^16–18^ Macrophages in tumor tissues, also called tumor-associated macrophages (TAMs), are the most abundant innate immune cells in the tumor microenvironment.^19,20^ Interestingly, ATMs might also be TAMs in the early phase of PC. M1 macrophages are effective in killing tumor cells by phagocytosis or mediating a T helper type 1 (Th1) response, while M2 macrophages support tumors by inducing immunosuppression and a Th2 response.^21,22^ Therefore, reprogramming from the M2 phenotype to the M1 phenotype is effective in cancer therapy, although TAMs are heterogeneous in phenotype.^23,24^

Nutrition management is an important part of comprehensive therapy for cancer patients.^25,26^ Metabolic reprogramming has been suggested to be a major characteristic and contributor of malignancies.^27–29^ Emerging studies indicate that metabolic reprogramming of immune cells can also regulate antitumor immunity.^30–33^ Given that ATM activity can be regulated by HFD consumption,^17,34^ we presumed that HFD treatment might be a potential strategy for the modulation of the tumor microenvironment in CRC-PC, a disease with limited intervention strategies. Thus, in the present study, we mimicked the seeding and growth processes of CRC-PC by intraperitoneal inoculation of CRC cells into mice, investigated the mechanisms of PC development, and finally proposed HFD-based preventive strategies for CRC-PC.

## Results

### HFD suppresses the metastatic seeding of CRC

**Fig. 1:**
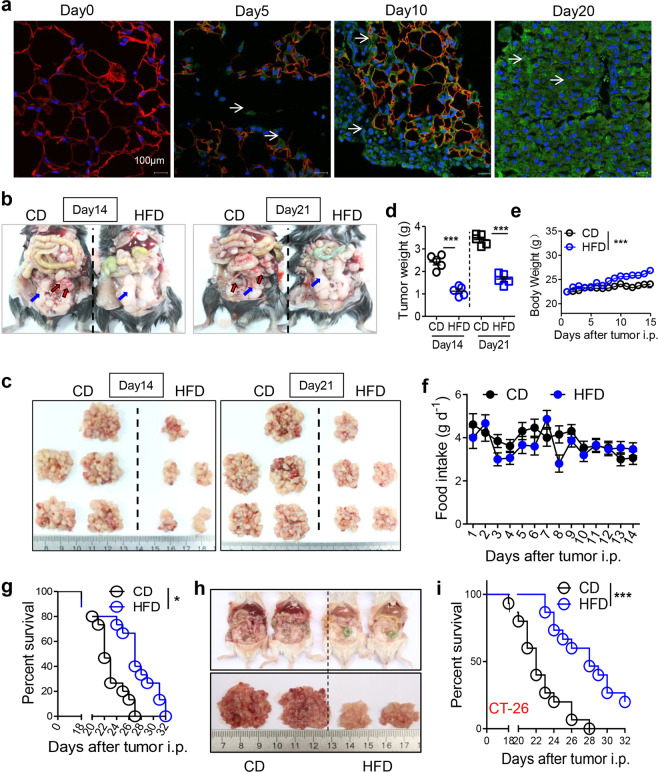
High-fat diet suppresses the metastatic seeding of CRC cells. **a** Metastatic seeding of CRC cells in the epididymal fat (eFat). Six-week-old male mice were intraperitoneally injected with GFP-tagged MC-38 cells (1.0 × 10^6^ cells in 100 µl PBS per mouse) or PBS, which was used as a control. The eFats were collected dynamically and stained with anti-GFP (green) or anti-perilipin (red) antibodies for immunofluorescence analysis. Nuclei were visualized by Hoechst staining (blue). Representative images are shown. Arrows indicate the cancer cells. Scale bars, 100 μm. **b** High-fat diet (HFD) feeding inhibits tumor seeding in the eFats. Six-week-old male mice were intraperitoneally inoculated with MC-38 cells (1.0 × 10^6^ cells in 100 µl PBS per mouse) and immediately fed a chow diet (CD) or HFD for 14 or 21 days. Then the tumor nodes in the eFats were evaluated. The blue and red arrows indicate the epididymal fat pads and tumor nodes, respectively. **c** Tumor nodes from the mice described above in **b**. Representative results for one mouse are shown. **d** Weights of the tumor nodes described in **c**. Data are shown as the mean ± s.e.m. (*n* = 5, ***P < 0.005; Student’s *t* test). **e** Dynamic measurement of the weights of the tumor-bearing mice described in **b**. Data are shown as the mean ± s.e.m. (*n* = 10, ***P < 0.005; two-way ANOVA). **f** Dynamic measurement of the food intake of the tumor-bearing mice described in **b**. Each plot represents the average food intake by one mouse in 1 day (*n* = 10; two-way ANOVA). **g** HFD consumption improves the survival of tumor-seeded mice. Tumor-seeded models were established as described in **b**. This experiment was repeated three times (*n* = 15, *P < 0.05; Gehan–Breslow–Wilcoxon test). **h** HFD inhibits the peritoneal seeding of CT-26 cells. Six-week-old male mice were intraperitoneally implanted with CT-26 cells (1.0 × 10^6^ cells in 100 µl PBS) and immediately fed a CD or HFD for 14 days. Then peritoneal tumor nodes were identified. **i** HFD consumption improves the survival of the tumor-seeded mice described in **h**. (*n* = 15, ***P < 0.005; Gehan–Breslow–Wilcoxon test)

To investigate the role of HFD consumption in CRC metastasis, we successfully established a mouse model mimicking peritoneal metastasis by intraperitoneally injecting the CRC cell line MC-38 or CT-26. We revealed that CRC cells preferentially and quickly migrated to the visceral fat (Fig. 1a and Fig. S1a). Almost all the visceral fat was exhausted 20 days post tumor injection in our experimental condition (Fig. 1a). Unexpectedly, HFD consumption obviously inhibited tumor formation in the epididymal fats (eFats; Fig. 1b–d), the mesenterium (Fig. S1b), the omentum majus (Fig. S1c), and the perirenal fat (Fig. S1d) on day 14 and day 21 after tumor injection. Simultaneously, HFD consumption obviously increased body weight, while food intake was unchanged (Fig. 1e, f). Importantly, the survival of mice with peritoneal metastasis was markedly improved by HFD treatment (Fig. 1g). These antitumor effects of the HFD were confirmed in another peritoneal metastasis model established with CT-26 cells (Fig. 1h, i and Fig. S2a–c). In addition, we verified that HFD consumption suppressed lung metastasis by MC-38 cells in an intravenous metastasis model (Fig. S2d). However, the growth of MC-38 cells in a subcutaneous (s.c.) tumor model was not influenced by HFD treatment (Fig. S2e). These results indicated that the HFD only affected the metastasis, not the growth, of CRC cells. Therefore, a HFD might only affect the early phase of CRC metastasis. As expected, we demonstrated that timely treatment with a HFD for 7 days was sufficient to improve the survival of mice with peritoneal metastasis. In contrast, delaying HFD treatment did not benefit survival (Fig. S2f). These results indicated that HFD consumption could suppress the metastatic seeding of CRC cells.

We next established another three tumor-seeded mouse models with preexisting primary tumors at different sites to observe whether the primary tumors would affect the therapeutic effect of HFD consumption. We found that HFD consumption did not affect the growth of primary tumors in s.c., in situ or Apc^min+/^^−^ tumor-seeded models (Fig. S3a, c, e). However, HFD consumption markedly suppressed the metastatic seeding of MC-38 cells in these models (Fig. S3b, d, f). These results indicated that the preexisting primary tumors did not affect the therapeutic effect of HFD consumption on the metastatic seeding of CRC cells. Thus we did not need to pre-establish primary tumors in subsequent studies.

### HFD potentiates the accumulation and activation of T cells in tumor-seeded eFats

**Fig. 2:**
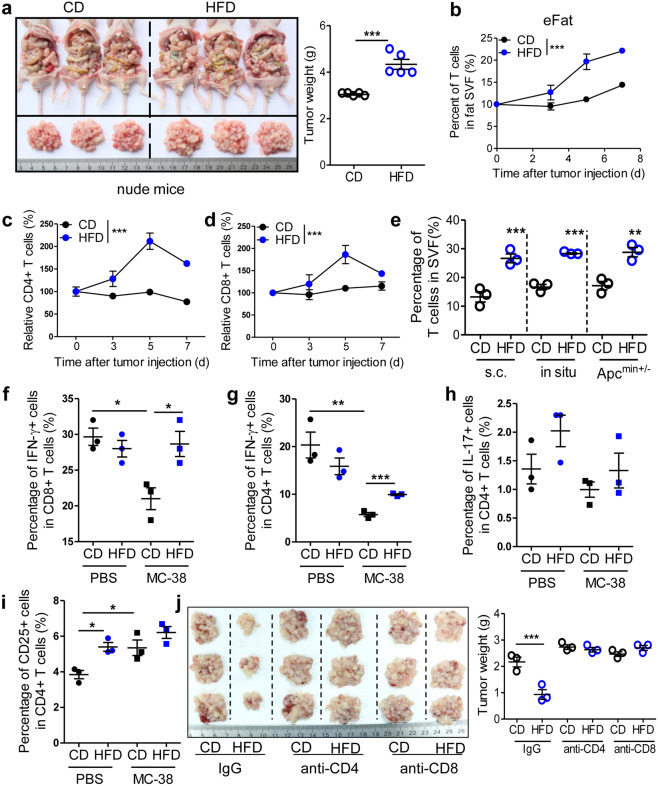
HFD potentiates T cell recruitment and activation in tumor-seeded eFats. **a** HFD consumption promotes the peritoneal metastasis of MC-38 cells in nude mice. Six-week-old male nude mice were intraperitoneally injected with MC-38 cells (1.0 × 10^6^ cells in 100 µl PBS) and immediately fed a CD or HFD for 14 days. Peritoneal tumor nodes were collected and weighed (*n* = 5). **b**–**d** HFD consumption enhances T cell infiltration into the eFats. Six-week-old male mice were intraperitoneally injected with MC-38 cells (1.0 × 10^6^ cells in 100 µl PBS) and immediately fed a CD or HFD. Then the stromal cells of the eFats were isolated dynamically. Total T cells (CD45^+^CD3^+^CD11b^−^CD45R^−^) (**b**), CD4^+^ T cells (CD45^+^CD3^+^CD11b^−^CD45R^-^CD4^+^CD8^−^) (**c**), and CD8^+^ T cells (CD45^+^CD3^+^CD11b^-^CD45R^-^CD4^-^CD8^+^) (**d**) were assessed by flow cytometry. Each tested sample was pooled from 4 individual samples (*n* = 3). **e** HFD consumption increases the frequencies of adipose tissue T cells in the s.c., in situ and Apc^min+/−^ tumor-seeded models described in “Methods.” Each tested sample was pooled from 2 individual samples (*n* = 3). **f** Frequencies of IFNγ^+^ cells among CD8^+^ T cells in the eFats of tumor-seeded mice treated with a CD or HFD for 5 days (*n* = 3). **g**–**i** Frequencies of IFNγ^+^ (**g**), IL17^+^ (**h**), and CD25^+^ (**i**) cells among CD4^+^ T cells in the eFats of tumor-seeded mice treated with a CD or HFD for 5 days (*n* = 3). **j** Six-week-old male C57BL/6 mice were intraperitoneally injected with MC-38 cells (1.0 × 10^6^ cells in 100 µl PBS) and immediately fed a CD or HFD for 14 days. Each mouse was intraperitoneally treated with antibodies (IgG, anti-CD4, or anti-CD8) 3 times (days 2, 4, and 6) at a dosage of 100 µg day^−1^. Peritoneal tumor nodes were collected and weighed. (*n* = 3). All the data are shown as the mean ± s.e.m. The data in **b**–**d** were analyzed with two-way ANOVA. The data in other histograms were analyzed with Student’s *t* test (*P < 0.05, **P < 0.01, and ***P < 0.005)

Unexpectedly, the inhibitory effect of HFD consumption on the peritoneal seeding of CRC cells was reversed in nude mice (Fig. 2a), which are deficient in lymphocytes. This result indicated that the HFD-mediated suppression of the metastatic seeding of CRC cells depended on lymphocytes. Thus we next observed the infiltration and activity of T cells during the process of tumor seeding. We demonstrated that HFD consumption obviously increased the frequencies of total, CD4^+^, and CD8^+^ T cells in tumor-seeded eFats (Fig. 2b–d and Fig. S4a–c). This increase in the intra-eFat T cell frequency was confirmed in the s.c., in situ and Apc^min+/−^ tumor-seeded models (Fig. 2e). Furthermore, we found that the frequencies of total, CD4^+^, and CD8^+^ T cells in the spleen were not altered in response to HFD treatment in tumor-seeded mice (Fig. S5a–f). However, the percentages of blood T cells declined in the HFD-treated group (Fig. S6a–f). These results indicated that HFD consumption might trigger T cell migration from the blood into the visceral fat.

In addition, we showed that the frequencies of IFNγ^+^CD4^+^ and IFNγ^+^CD8^+^ T cells were increased by HFD consumption, while the frequencies of IL-17^+^CD4^+^ and CD25^+^CD4^+^ T cells were not altered in response to the HFD (Fig. 2f–i and Fig. S7). Consistently, HFD consumption stimulated a proinflammatory environment in tumor-seeded eFats (Fig. S8a, b). Functionally, we showed that treatment with anti-CD4 or anti-CD8 antibodies fully prevented the HFD-mediated suppression of CRC seeding (Fig. 2j).

### HFD stimulates C-X-C chemokine motif ligand 10 (Cxcl10)-dependent T cell recruitment in tumor-seeded eFats

**Fig. 3:**
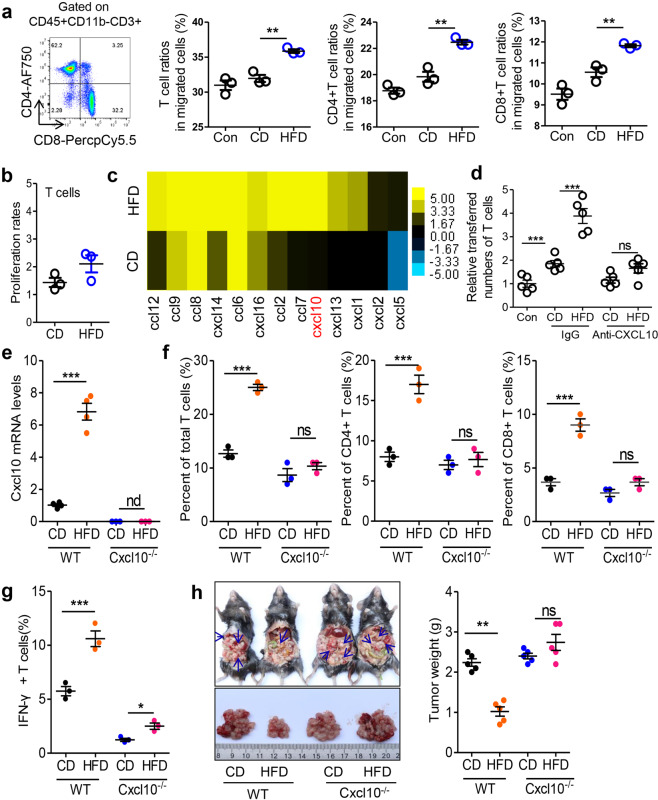
HFD stimulates Cxcl10-dependent T cell recruitment in tumor-seeded eFats. **a** The eFats of HFD-treated mice attracted T cells. The eFats were isolated from tumor-seeded mice fed a CD or HFD for 5 days. Mouse splenocytes were cultured in the upper chamber, and the eFats were cultured in the lower chamber of a Transwell system. Twenty-four hours later, the migrated T cells, CD4^+^ T cells, and CD8^+^ T cells were analyzed by flow cytometry (*n* = 3). **b** Proliferation rate of T cells cocultured with the eFats from tumor-seeded mice treated with a CD or HFD for 5 days (*n* = 5). **c** Heat map showing differentially expressed chemokines in the eFats. mRNA sequencing assays were performed on the eFats isolated from tumor-seeded mice fed a CD or HFD for 5 days. Each sample was pooled from three individual samples. **d** CXCL10 is required for HFD-induced T cell migration ex vivo. The eFats were isolated from tumor-seeded mice fed a CD or HFD for 5 days. Mouse splenocytes were cultured in the upper chamber, and the eFats were cultured in the lower chamber of a Transwell system. A neutralizing anti-CXCL10 antibody (100 ng ml^−1^) was added into the lower chamber to block CXCL10 activity. Twenty-four hours later, the migrated T cells in the lower chamber were analyzed by flow cytometry. T cells were defined as CD45^+^CD11b^−^CD3^+^ (*n* = 5). **e** Relative mRNA levels of Cxcl10 in the eFats from tumor-seeded WT or Cxcl10 knockout (Cxcl10^−/−^) mice fed a CD or HFD for 5 days. nd not detected (*n* = 4). **f**, **g** Infiltration of total T cells, CD4^+^ T cells, CD8^+^ T cells (**f**), and IFNγ^+^ T cells (**g**) into the eFats of WT or Cxcl10^−/−^ mice fed a CD or HFD for 5 days. Each sample was pooled from 3 individual samples (*n* = 3). **h** An MC-38-based tumor-seeded model was constructed in WT and Cxcl10^−/−^ mice. Peritoneal tumor nodes were collected on day 14 and weighed (*n* = 5). All data are shown as the mean ± s.e.m. (*P < 0.05, **P < 0.01 and ***P < 0.005; ns not significant; Student’s *t* test)

The increase in the T cell frequency might result from increased recruitment or proliferation. Thus we next investigated whether HFD-primed tumor-seeded eFats affect T cell migration or proliferation. The results showed that HFD-primed eFats promoted T cell migration (Fig. 3a), while the proliferation of T cells remained unchanged (Fig. 3b). To explore which factor is involved in T cell migration in this context, a series of HFD-associated chemokines including ccl12, ccl9, ccl8, ccl7, ccl6, ccl2, cxcl16, cxcl14, cxcl13, cxcl10, cxcl5, cxcl2, and cxcl1 were screened by RNA sequencing analysis (Fig. 3c). Among these chemokines, CXCL10,^35^ CXCL16,^36^ CCL8,^37^ and CCL2^38^ are reported to be associated with T cell infiltration and function. In addition, CXCL10 induction by HFD was the most obvious among the four T cell migration-associated chemokines (Fig. 3c). We next demonstrated that blockade of CXCL10 (but not CCL2) with antibodies prevented HFD-induced T cell migration to the eFats ex vivo (Fig. 3d and Fig. S8c). To confirm the function of Cxcl10 in eFat T cell recruitment, Cxcl10 knockout (Cxcl10^−/−^) mice were employed. We showed that HFD-stimulated Cxcl10 expression in the eFats was lost in Cxcl10^−/−^ mice (Fig. 3e). Simultaneously, the promotive effects of HFD consumption on the infiltration of total, CD4^+^, and CD8^+^ T cells disappeared (Fig. 3f). However, the HFD could still increase the frequency of IFNγ^+^ T cells, although the total number of IFNγ^+^ T cells was largely reduced in Cxcl10^−/−^ mice (Fig. 3g). Functionally, the HFD-mediated suppression of peritoneal seeding was also lost in the context of Cxcl10 deletion (Fig. 3h).

### HFD potentiates macrophage-dependent T cell recruitment and activation in tumor-seeded eFats

**Fig. 4:**
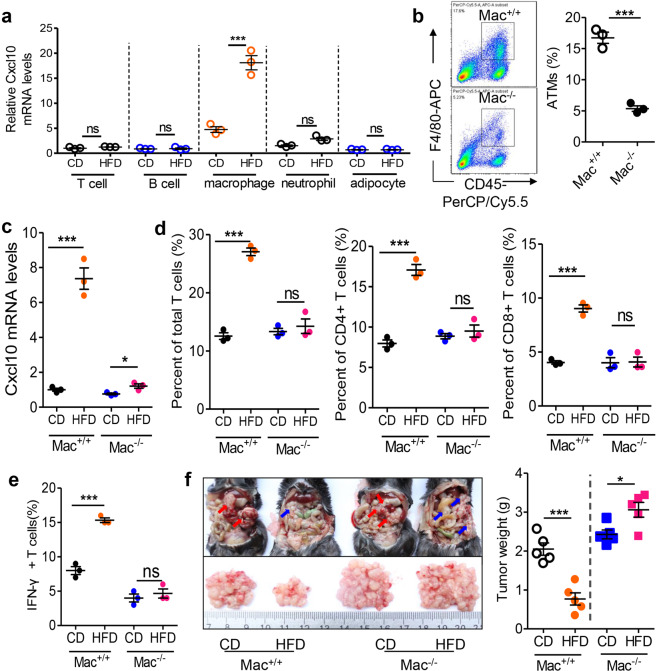
HFD potentiates macrophage-dependent T cell recruitment and activation in tumor-seeded eFats. **a** Relative Cxcl10 mRNA levels in T cells, B cells, macrophages, neutrophils, and adipocytes isolated from the eFats of tumor-seeded mice treated with a CD or HFD for 5 days. Each sample was pooled from 10 individuals (*n* = 3). **b** Adipose tissue macrophages (ATMs) in the eFats were depleted in Mac^−/−^ mice. Details are described in “Methods” (*n* = 3). **c** Relative mRNA levels of Cxcl10 in the eFats of tumor-seeded Mac^+/+^ or Mac^−/−^ mice fed a CD or HFD for 5 days (*n* = 3). **d** HFD consumption stimulates macrophage-dependent T cell recruitment. Six-week-old male Mac^+/+^ and Mac^−/−^ mice were intraperitoneally injected with MC-38 cells (1.0 × 10^6^ cells in 100 µl PBS) and immediately fed a CD or HFD for 5 days. Then the frequencies of total T cells, CD4^+^ T cells, and CD8^+^ T cells were calculated by flow cytometry. Each tested sample was pooled from 3 individual samples (*n* = 3). **e** Macrophage deletion prevents HFD-induced T cell activation. IFNγ^+^ T cells were counted in the eFats of tumor-seeded mice treated with a CD or HFD for 5 days. Each tested sample was pooled from 3 individual samples (*n* = 3). **f** Macrophage deletion prevents the HFD-mediated suppression of tumor seeding. Mac^+/+^ and Mac^−/−^ mice were intraperitoneally injected with MC-38 cells (1.0 × 10^6^ cells in 100 µl PBS) and immediately fed a CD or HFD for 14 days. Peritoneal tumor nodes were collected and weighed (*n* = 5). All the data are shown as the mean ± s.e.m. (*P < 0.05 and ***P < 0.005; ns not significant; Student’s *t* test)

To explore the sources of Cxcl10 in tumor-seeded eFats, we sorted the stromal cells in the eFats and measured the mRNA levels of Cxcl10 in response to HFD treatment. The results showed that macrophages had obviously higher levels of Cxcl10 than T cells, B cells, neutrophils, and adipocytes (Fig. 4a). We next employed myeloid cell-specific deletion (Mac^−/−^) mice (Fig. 4b) and found that HFD-stimulated Cxcl10 expression in tumor-seeded eFats was almost completely lost (Fig. 4c). Accordingly, the HFD-induced infiltration of total, CD4^+^, CD8^+^ (Fig. 4d), and IFNγ^+^ (Fig. 4e) T cells was totally prevented by macrophage deletion. The inhibitory effect of HFD consumption on metastatic seeding was not observed in Mac^−/−^ mice (Fig. 4f).

### Stearic acid stimulates macrophage activity in vitro via toll-like receptor 4 (TLR4)

**Fig. 5:**
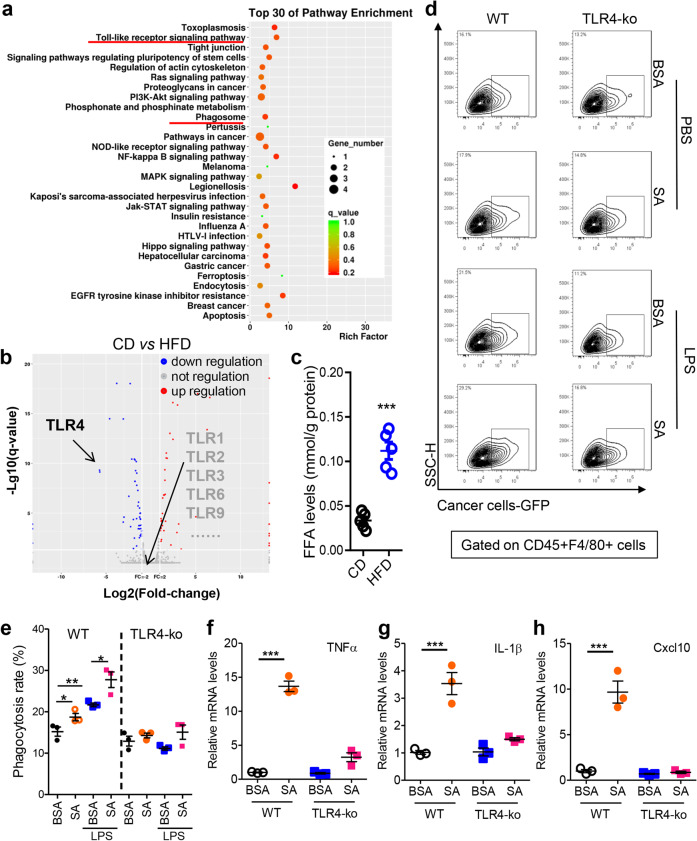
Stearic acid stimulates macrophage activation in vitro via TLR4. **a** The Toll-like receptor signaling pathway was highly enriched in the eFats of tumor-seeded mice treated with a CD or HFD for 5 days. Six-week-old male mice were intraperitoneally injected with MC-38 cells (1.0 × 10^6^ cells in 100 µl PBS) and immediately fed a CD or HFD for 5 days. Then the eFats were collected for RNA sequencing assays, and altered gene expression was subjected to KEGG pathway enrichment analysis. **b** Fold changes in gene expression determined by comparing RNA sequencing results between the CD group and the HFD group. Red points indicate upregulated genes; blue points indicate downregulated genes; gray points show unchanged genes. Toll-like receptor 4 (TLR4) expression was obviously upregulated by HFD consumption. **c** The level of free fatty acid (FFA) in eFats of the mice treated with CD or HFD for 5 days. **d** Stearic acid and LPS stimulate TLR4-dependent phagocytosis in macrophages. Peritoneal macrophages from wild-type (WT) or TLR4 knockout (TLR4-ko) mice were pretreated with stearic acid (SA; 200 µM) and/or LPS (100 ng ml^−1^) for 12 h and cocultured with GFP-tagged MC-38 cells for phagocytosis assays. This experiment was repeated twice. Representative results are displayed. **e** Statistical analysis of the results in **d**. Data are presented as the mean ± s.e.m. (*n* = 3; *P < 0.05 and **P < 0.01; Student’s *t* test). **f**–**h** A fatty acid stimulates TLR4-dependent proinflammatory cytokine expression. Peritoneal macrophages from WT or TLR4-ko mice were treated with SA (200 µM) or LPS (100 ng ml^−1^) for 6 h, and then RNA was extracted for real-time PCR analysis of TNFα, IL-1β, and Cxcl10. Data are presented as the mean ± s.e.m. (*n* = 3; ***P < 0.005; Student’s *t* test)

The aforementioned results showed that macrophages promoted not only the recruitment but also the activation of T cells in tumor-seeded eFats, which implies that macrophages in the eFats are also activated by HFD consumption. Indeed, HFD consumption increased the frequencies of M1-like macrophages in the eFats in the s.c., in situ and Apc^min+/−^ tumor-seeded models (Fig. S9a–d). In addition, the expression of macrophage Sirpα, a negative checkpoint of phagocytosis,^39^ was induced by seeding tumors and was suppressed by HFD treatment (Fig. S9e). According to RNA sequencing and KEGG enrichment analyses, TLR- and phagosome-related signaling pathways in the eFats were highly enriched in response to HFD treatment (Fig. 5a). Differential gene expression analysis showed that TLR4 was markedly induced by HFD treatment (Fig. 5b). Saturated free fatty acids and lipopolysaccharides (LPS) are commonly used as stimulators to mimic HFD-associated inflammation.^17,34^ Indeed, we verified that the level of free fatty acid in eFats was induced by a HFD for 5 days (Fig. 5c). We next demonstrated that stearic acid and/or LPS induced phagocytosis by macrophages and these effects were abolished by knocking out TLR4 (Fig. 5d, e and Fig. S9f). Likewise, the expression of tumor necrosis factor-α (TNFα), interleukin (IL)-1β, and Cxcl10 induced by stearic acid was prevented by the deletion of TLR4 in macrophages (Fig. 5f–h).

### TLR4 is required for HFD-regulated macrophage activation and T cell recruitment and activation as well as metastatic seeding

**Fig. 6:**
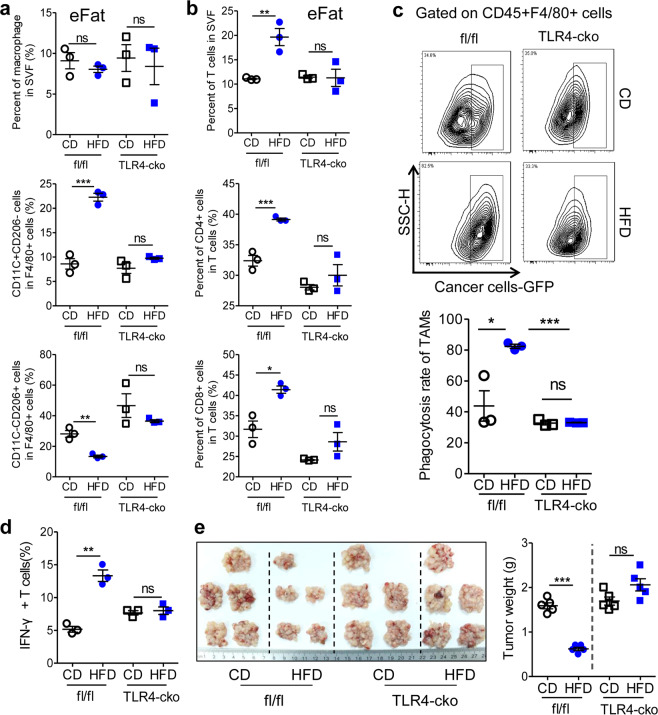
TLR4 is required for HFD-regulated macrophage activation and T cell recruitment and activation as well as peritoneal metastasis. **a** HFD consumption activates M1-like macrophages in the visceral fat via TLR4. Six-week-old male fl/fl and macrophage-specific TLR4 knockout (TLR4-cko) mice were intraperitoneally injected with MC-38 cells (1.0 × 10^6^ cells in 100 µl PBS) and immediately fed a CD or HFD for 5 days. Then total macrophages, M1 macrophages and M2 macrophages in the eFats were analyzed. Each tested sample was pooled from 3 individual samples (*n* = 3). **b** HFD consumption potentiates T cell recruitment via macrophage TLR4. Frequencies of total T cells, CD4^+^ T cells, and CD8^+^ T cells in the eFats of the mice described in **a** (*n* = 3). **c** HFD consumption stimulates tumor phagocytosis by macrophages via TLR4. fl/fl and TLR4-cko mice were injected with GFP-tagged MC-38 cells (1.0 × 10^6^ cells in 100 µl PBS). Fourteen days later, the in vivo phagocytosis rates of tumor-associated macrophages were measured (*n* = 3). **d** IFNγ^+^ T cells in the eFats of fl/fl or TLR4-cko mice fed a CD or HFD for 5 days. Each sample was pooled from 3 individual samples (*n* = 3). **e** HFD consumption suppresses tumor seeding via TLR4. MC-38-based tumor-seeded models were constructed in fl/fl and TLR4-cko mice. Fourteen days later, peritoneal tumor nodes were collected and weighed (*n* = 5). The data are shown as the mean ± s.e.m. (*P < 0.05, **P < 0.01, and ***P < 0.005; ns not significant; Student’s *t* test)

To validate the functions of TLR4 in HFD-associated macrophage activation and tumor regression in vivo, myeloid cell-specific TLR4 knockout (TLR4-cko) mice were employed. We demonstrated that HFD consumption increased the frequency of M1-like macrophages and inhibited the accumulation of M2-like macrophages in a TLR4-dependent manner in tumor-seeded eFats; however, the total number of macrophages in the eFats was not altered (Fig. 6a and Fig. S9a). Consistently, the HFD-stimulated infiltration of total, CD4^+^, and CD8^+^ T cells into tumor-seeded eFats was prevented in TLR4-cko mice (Fig. 6b). Furthermore, we validated that HFD-stimulated macrophage phagocytosis (Fig. 6c and Fig. S9g) and T cell activation (Fig. 6d) were fully abolished in TLR4-cko mice. The suppressive effect of HFD consumption on the metastatic seeding of CRC cells was not observed in TLR4-cko mice (Fig. 6e).

### HFD synergistically potentiates the therapeutic effects of chemotherapeutic drugs on peritoneal metastasis

**Fig. 7:**
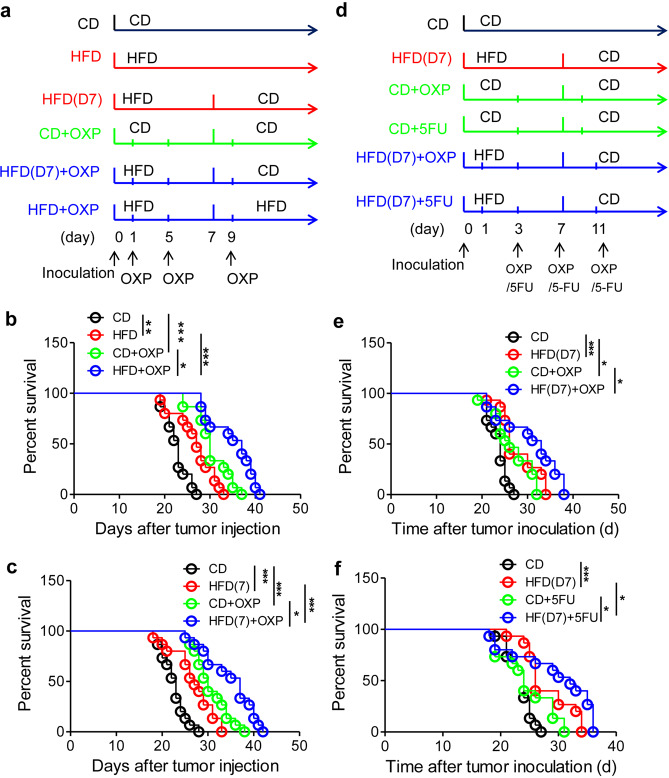
HFD synergistically potentiates the preventive effect of chemotherapeutic drugs on peritoneal metastasis. **a** Schematic diagram for the time points of diet and drug treatments. After tumor inoculation, mice were organized into six groups with different diets and oxaliplatin treatments. CD group: mice were treated with a CD the entire time; HFD group: mice were treated with a HFD the entire time; HFD(7) group: mice were treated with the HFD from day 1 to day 7; CD + OXP group: mice were treated with the CD the entire time and oxaliplatin (5.0 mg kg^−1^) on days 1, 5, and 9 after tumor inoculation; HFD + OXP group: mice were treated with the HFD the entire time and oxaliplatin (5.0 mg kg^−1^) on days 1, 5, and 9 after tumor injection; and HFD(7)+OXP group: mice were treated with the HFD from day 1 to day 7 and oxaliplatin (5.0 mg kg^−1^) on days 1, 5, and 9 after tumor injection. **b** Long-term HFD consumption synergistically potentiates the survival of oxaliplatin-treated tumor-seeded mice. Mice were grouped and treated as described in **a**. The survival times of these tumor-bearing mice were recorded. **c** Seven days of HFD feeding synergistically potentiated the survival of oxaliplatin-treated tumor-seeded mice. Mice were treated as described in **a**. **d** Schematic diagram for the time points of diet and drug treatments. After tumor inoculation on day 0, mice were organized into 6 groups with different diets and drug treatments. CD group: mice were treated with a CD the entire time; HFD(7) group: mice were treated with a HFD from day 1 to day 7; CD + OXP group: mice were treated with the CD the entire time and oxaliplatin (2.5 mg kg^−1^) on days 3, 7, and 11; CD + 5FU group: mice were treated with the CD the entire time and 5-fluorouracil (25 mg kg^−1^) on days 3, 7, and 11; HFD(7) + OXP group: mice were treated with the HFD from day 1 to day 7 and oxaliplatin (2.5 mg kg^−1^) on days 3, 7, and 11; and HFD(7)+5FU group: mice were treated with the HFD from day 1 to day 7 and 5-fluorouracil (25 mg kg^−1^) on days 3, 7, and 11. **e** HFD feeding and oxaliplatin synergistically potentiate the survival of the tumor-seeded mice described in **d**. **f** HFD and 5-fluorouracil synergistically potentiate the survival of the tumor-seeded mice described in **d**. All the data are shown as the mean ± s.e.m. (*n* = 15; *P < 0.05, **P < 0.01, and ***P < 0.005; Gehan–Breslow–Wilcoxon test)

We next observed whether the peritoneal metastasis of CRC can be prevented by combined treatment with a HFD and other chemotherapeutic drugs. OXP and 5FU are commonly used to treat advanced CRC.^5^ We demonstrated that OXP largely improved the survival of tumor-seeded mice, and this effect was obviously potentiated by the addition of a HFD over the whole course (Fig. 7a, b). Notably, HFD treatment for only 7 days in the early phase achieved a benefit similar to that conferred by long-term treatment (Fig. 7a–c). We next halved the OXP dose and delayed treatment to reduce the side effects of chemotherapy. We demonstrated that the therapeutic effect of HFD feeding for 7 days was comparable to that of OXP. We could still observe the synergistic effects of HFD feeding on OXP treatment (Fig. 7d, e). In addition, the synergistic effect of HFD feeding on 5FU-based treatment of metastatic seeding was also verified (Fig. 7d, f). More directly, we showed that HFD and chemotherapeutic drugs (OXP and 5FU) had a synergistic effect in suppressing peritoneal seeding of CRC cells (Fig. S10a, b). These findings indicated that a HFD could be used as an adjuvant to regular chemotherapy in treating the metastatic seeding of CRC.

## Discussion

**Fig. 8:**
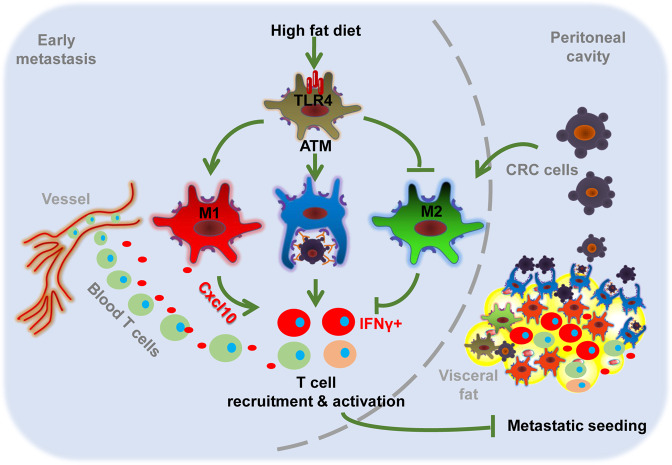
Proposed hypothesis for the prevention of colorectal peritoneal metastasis by a HFD. Disseminated colorectal cancer cells preferentially target the visceral fat in early metastasis. HFD consumption stimulates TLR4-dependent M1 macrophage activation and phagocytosis in adipose tissue macrophages (ATMs), which further enhance the recruitment (by Cxcl10) and activation (by M1 cytokines) of CD4+ and CD8+ T cells in the visceral fat and ultimately prevents the metastatic seeding of colorectal cancer

In addition to patients in the terminal phase, patients treated with curative surgery frequently experience PC due to the peritoneal dissemination of CRC cells via the ruptured vasculature.^2^ However, there are no effective strategies for the prevention of this condition, resulting in an extremely low survival rate in the affected patients. Thus preventive strategies for CRC-PC appear rather important. With multiple metastatic models established in mice, we revealed an unexpected role for a HFD in preventing the metastatic seeding of CRC cells. We demonstrated that both innate and adaptive immunity were involved in the antitumor process of HFD treatment. The TLR4–Cxcl10 axis in ATMs/TAMs played a central role in the recruitment of T cells, and TLR4-mediated M1 macrophage activation was critical for T cell activation in tumor-seeded eFats (Fig. 8). Our findings might provide a potential strategy for the management of CRC-PC patients in the clinic.

It should be noted that HFD consumption might have two aspects in regulating cancer biology. The conventional opinion is that obesity potentiates the occurrence and development of multiple cancers.^40,41^ Here we reported a seemingly contradictory effect of HFD consumption suppressing tumor seeding. Actually, we should distinguish between HFD consumption and obesity. Increased dietary fat consumption for a short time would not induce obesity or metabolic syndrome.^17,34^ Notably, the antitumor effect of HFD treatment relied on innate and adaptive immunity, without which the HFD might promote tumor progression (Fig. 2a and Fig. 4f) by providing sufficient energy to cancer cells. Therefore, HFD treatment should be provided for only a short period to avoid inducing obesity, and patients need to have a certain immune profile for selection. If the patients were in cachexy with extremely low immunity, HFD might not stimulate enough immunity to kill the invading tumor cells. So we highly recommended the HFD treatment in the surgery patients, who are usually in early or middle stages of CRC. Those patients should have enough basic immune profile. Simultaneously, the operation-resulted cancer cell dissemination also need to be timely cleared.

Our findings indicated that the timeliness of HFD treatment was critically important. Tumor-seeded mice that consumed a HFD for 7 days in the early phase achieved a benefit similar to that in those receiving the HFD for the whole course. This finding largely indicated the potential for application of HFD treatment in surgery patients with CRC. It should be pointed out that an optimal HFD treatment must occur closely after surgery because a large amount of cancer cells might be released and spread rapidly in this time window. Coincidently, fat emulsion is routinely used on the CRC patients after surgery for providing energy. However, whether fat emulsion after surgery prevents PC in patients requires further observation in a large clinical trial. In addition, for CRC patients in advanced stages without PC, intermittent HFD treatment might also potentiate the antitumor response in adipose tissues and ultimately prevent PC onset because our studies indicated that the therapeutic effect of HFD consumption on PC was not influenced by preexisting primary tumors. Consuming a meal high in fat is safer and more economical than any chemotherapeutic drug. Thus HFD treatment might be widely applicable in the management of CRC-PC.

It should be pointed out that HFD consumption could be combined with other drugs for the prevention of CRC-PC. CRC-PC mice receiving combined treatment with a HFD and OXP or 5FU achieved better survival than those treated with either drug alone. There are several other drugs, such as leucovorin and irinotecan, which are used in the treatment of CRC-PC.^42^ When combined with HFD treatment, the dose of these drugs could be reduced, and side effects could also be limited.

Overall, we claimed for the first time that HFD treatment in the early phase could prevent the metastatic seeding of CRC through TLR4-dependent activation of innate and adaptive immunity. Our findings represent a novel concept and potential strategy for preventing CRC-PC in the clinic.

## Materials and methods

### Cell culture

The mouse CRC cell lines MC-38, CT-26, and MC-38G (green fluorescent protein (GFP)-tagged MC-38 cells) were maintained in our laboratory.^10,33^ All cells were authenticated and tested for mycoplasma. Primary mouse macrophages and cell lines were cultured in regular medium (Dulbecco’s modified Eagle’s medium (DMEM) supplemented with 10% fetal bovine serum (FBS)) at 37 °C in a humidified 5% CO_2_ atmosphere.

### Mice

Mouse studies were approved by the Institutional Animal Care and Use Committee of Third Military Medical University (TMMU) and were carried out according to relevant guidelines. All mice were housed in a pathogen-free facility with a 12-h light/12-h dark cycle in TMMU. All mice were provided food and purified water ad libitum. Wild-type C57BL/6 and BALB/c mice and BALB/c nude mice were provided by TMMU. C57BL/10ScNJNju mice (#000192) with global knockout of the *tlr4* gene were obtained from the National Model Animal Resource Information Platform (Nanjing University, China).^43,44^ ROSA26-eGFP-DTA mice (#006331, Jackson Laboratory Stock) have a loxP-flanked STOP cassette preventing the expression of diphtheria toxin fragment A (DTA). Exposure to Cre recombinase removes the lox-stop fragment, resulting in ablation of cre-expressing cells. These ROSA26-eGFP-DTA mice are useful for Cre-induced deletion of specific groups of cells.^45^ Myeloid cell-specific deletion (Mac^−/−^) mice were generated by crossing ROSA26-eGFP-DTA mice with mice expressing lysozyme promoter-driven Cre recombinase (#004781, Jackson Laboratory Stock). Myeloid cell-specific TLR4 knockout (TLR4-cko) mice were obtained by crossing B6(Cg)-Tlr4tm1.1Karp/J mice (#024872, Jackson Laboratory Stock) with mice expressing lysozyme promoter-driven Cre recombinase (#004781, Jackson Laboratory Stock). B6.129S4-Cxcl10^tm1Adl^/J mice with Cxcl10 knocked out (Cxcl10^−/−^)^35^ were provided by The Jackson Laboratory (#006087). The APC^min+/−^ mice were generously provided by Professor Wenhui Ma in Southern Medical University in Guangzhou (China).

### Mouse models for peritoneal metastasis of CRC

To mimic the peritoneal dissemination of CRC cells, the MC-38 cells or CT-26 cells (1.0 × 10^6^ cells in 0.1 ml phosphate-buffered saline (PBS) for each mouse) were intraperitoneally injected into abdominal cavity of six-week-old male C57BL/6 or BALB/c mice, respectively. Then the tumor-inoculated mice were immediately fed with a low-fat chow diet (#D12450B, fat content 10% by calorie, Research Diets) or HFD (#D12492, fat content 60% by calorie, Research Diets). Mice were sacrificed on day 14 or 21 for observing the peritoneal seeding nodes. To observe the alteration of immune cells in visceral fats, spleens or blood, the tumor-bearing mice were sacrificed on day 0, 3, 5, and 7. Those mice were also treated with HFD at different time periods, and survival time was recorded. This study was approved by the Institutional Animal Care and Use Committee of TMMU and was performed in accordance with relevant guidelines.

### Peritoneal metastasis models with preexisting primary tumors

To investigate whether preexisting primary tumors affect the impact of HFD consumption on peritoneal metastatic seeding, three different tumor-seeded models were established. (1) A s.c. tumor-seeded model: 6-week-old male mice were s.c. inoculated with MC-38 cells (1.0 × 10^6^ cells in 100 µl PBS) on day 0 and then intraperitoneally injected with MC-38 cells (1.0 × 10^6^ cells in 100 µl PBS) on day 7. (2) An in situ tumor-seeded model: 6-week-old male mice were inoculated with MC-38 cells in the subserosa of the cecum (1.0 × 10^6^ cells in 100 µl PBS) on day 0 and then intraperitoneally injected with MC-38 cells (1.0 × 10^6^ cells in 100 µl PBS) on day 7. (3) An Apc^min+/−^ tumor-seeded model: 3-month-old female Apc^min+/−^ mice were intraperitoneally inoculated with MC-38 cells (1.0 × 10^6^ cells in 100 µl PBS) on day 0.

All of these models were treated with a HFD on the day after peritoneal injection. Five days later, the eFats were isolated for the analysis of macrophages and T cells. Fourteen days later, the primary tumors and seeded nodes were evaluated by measuring volume, weight, or number.

### Mouse models for intravenous metastasis of CRC

Six-week-old male mice were intravenously injected with MC-38 cells (5.0 × 10^6^ cells in 0.1 ml PBS per mouse) via the tail vein. The mice were sacrificed 14 days after tumor inoculation. The lungs were dissected and weighed. This study was approved and performed in accordance with relevant guidelines of TMMU.

### Subcutaneous tumor models

Six-week-old male C57BL/6 mice were s.c. injected with MC-38 cells (5.0 × 10^6^ cells per mouse) in their thighs. The tumor volume (size) was measured dynamically and calculated as 0.523 × (length × width × height). This study was approved by the Institutional Animal Care and Use Committee of TMMU and was performed in accordance with relevant guidelines.

### Isolation of peritoneal macrophages (PMs)

Each mouse was injected with 3 ml of 3% thioglycollate (#T9032, Sigma) on day 0 and killed with isoflurane on day 3. After intraperitoneal injection of 5 ml DMEM cell culture medium containing 10% FBS, as well as penicillin and streptomycin, the peritoneal cells were collected in cell culture dishes. Two hours later, the floating cells were removed by washing the cells with PBS. The attached cells were considered to be PMs (purity: ~90%) and were subjected to further experiments.

### Immunofluorescence staining

All tissue samples were fixed and processed into paraffin blocks. After dewaxing, antigen repair (10 mmol l^−1^ sodium citrate for 5 min in a pressure cooker) and removing endogenous enzymes in hydrogen peroxide block, the sections were incubated with the primary antibodies anti-GFP (ab13970, Abcam, UK, the dilution ratio was 1:500) and anti-Perilipin (ab3526, Abcam, UK, the dilution ratio was 1:200) at 4 °C overnight. Then the samples were incubated in secondary antibody (703–545–155, Jackson ImmunoResearch, USA; ab150074, Abcam, UK, the dilution ratio was 1:500) for 2 h at room temperature in the dark. After rinsing three times in 1× PBS for 5 min each, the slides were counterstained with Hoechst (C1026, Beyotime, China, the dilution ratio was 1:500) to reveal cell nuclei. Finally, fluorescence microscopy was used to evaluate specimen.

### Cell preparation, immunostaining, and fluorescence-activated cell sorting (FACS)

Mice were sacrificed by CO_2_. The spleen was dissected and gently rubbed between the two rough sides of frosted slides (#FSL006, Beyotime, Shanghai, China). Splenic samples were isolated by gently lapping spleens over a 75-μm filter (F513442, Sangon Biotech, China) and then washing in FACS buffer (PBS with 0.5% endotoxin-free FBS, 2 mM EDTA, and 25 mM HEPES). After spinning down for 5 min at 500 × *g*, erythrocytes were removed by ACK lysis buffer (R1010, Solarbio, China) for 5 min. Then the reaction was stopped by adding equivoluminal PBS buffer, and splenocytes were collected by centrifugation. Finally, cell pellets were resuspended with FACS buffer.

Circulating blood samples were collected using blood collection tubes (171001, KANG JIAN, China) to prevent clotting. After using the Mouse Peripheral Blood Lymphatic Separation Kit (#P8620, Solarbio, China), cells were collected and lysed in ACK lysis buffer as described above. In the same way, blood samples were resuspended in FACS buffer.

Adipose tissues were resected from mice and mechanically dissociated with ophthalmic forceps into about 1 mm^3^ and then digested with 25 ml Roswell Park Memorial Institute (RPMI) 1640 medium, supplemented with 3% FBS and 1 mg ml^−1^ collagenase IV (A004186, Sangon Biotech, China) for 45 min at 37 °C. Meanwhile, the solution was vibrated by magnetic stirrer. After dissociation, adipose tissue suspensions were filtered through a 75-μm filter and were spun down for 10 min at 500 × *g*. Then adipose tissue samples were lysed with ACK buffer as above and then resuspended in FACS buffer.

The fresh tumor tissues were cut into pieces and digested in Buffer A containing 1 g l^−1^ type 4 collagenase (#LS004188, Worthington), 0.1 g l^−1^ hyaluronidase (#H1115000, Sigma), and 0.01 g l^−1^ DNase I (#D8071, Solarbio, China). The dissociated cells were collected into a 15-ml tube and centrifuged at 400 × *g* for 5 min. The pellets were resuspended with ACK Lysing Buffer and washed with Buffer A before filtration with a 75-μm filter. These cells were collected for further isolation of macrophages and other immune cells.

At last, after staining with antibodies at 4 °C for 45 min followed by 2 washes in FACS buffer, the cells were resuspended in FACS buffer for FACS analysis (FACSVerse, BD Biosciences). The antibodies included PerCP/Cy5.5 anti-mouse CD45 Antibody (#103132, BioLegend), allophycocyanin (APC) anti-mouse F4/80 Antibody (#123116, BioLegend), fluorescein isothiocyanate anti-mouse CD206 (MMR) Antibody (#141704, BioLegend), Alexa Fluor700 anti-mouse CD11c Antibody (#117320, BioLegend), phycoerythrin (PE) anti-mouse/human CD45R/B220 Antibody (#103208, BioLegend), PE anti-mouse CD3 Antibody (#100206, BioLegend), APC/Cy7 anti-mouse Ly-6G/Ly-6C (Gr-1) Antibody (#108424, BioLegend), PE anti-mouse CD172a (SIRPα) Antibody (#144011, BioLegend), Alexa Fluor® 488 anti-mouse CD45 Antibody (#103122, BioLegend), APC/Cy7 anti-mouse CD4 Antibody (#100414, BioLegend), PerCP/Cy5.5 anti-mouse CD8a Antibody (#100734, BioLegend), APC anti-mouse/human CD45R/B220 Antibody (#103211, BioLegend), and APC anti-mouse/human CD11b Antibody (#101212, BioLegend). Macrophages are assessed as CD45^+^F4/80^+^CD3^−^Gr1^−^. M1-like macrophages were marked as CD45^+^F4/80^+^CD3^−^Gr1^−^CD11c^+^CD206^−^. M2-like macrophages were assessed as CD45^+^F4/80^+^CD3^−^Gr1^−^CD11c^−^CD206^+^. T cells were assessed as CD45^+^CD3^+^CD11b^−^CD45R^−^. CD4^+^ T cells were marked as CD45^+^CD3^+^CD11b^−^CD45R^−^CD4^+^CD8^−^. CD8^+^ T cells were assessed as CD45^+^CD3^+^CD11b^−^CD45R^−^CD4^−^CD8^+^.

### In vitro phagocytosis assays

PMs were plated (5.0 × 10^5^ per well) in a 6-well tissue culture plate and incubated in serum-free medium for 2 h before 2 × 10^6^ MC-38G cells were added. After coculturing for 4 h at 37 °C, the cells were harvested. The PMs were stained with PerCP/Cy5.5-conjugated anti-CD45 and APC-conjugated anti-F4/80 antibodies, and flow cytometry was performed. A total of 40,000 cells in each sample were analyzed. Unstained control and single-stained cells were prepared for gating. PMs with GFP were considered phagocytizing cells.

### In vivo phagocytosis assays

Six-week-old male C57 mice were intraperitoneally engrafted with MC38G cells (1.0 × 10^6^ cells in 100 µl PBS per mouse). Seventeen days later, the peritoneal tumor nodes were resected, mechanically dissociated with ophthalmic forceps into approximately 1-mm^3^ pieces and then digested with 25 ml HBSS supplemented with 1 g l^−1^ collagenase IV (A004186, Sangon Biotech, China) and 0.01 g l^−1^ DNase I (# D8071, Solarbio, China) for 1 h at 37 °C. Meanwhile, the solution was agitated with a magnetic stirrer. After dissociation, the tumor suspensions were filtered through a 75-μm filter and spun down for 10 min at 500 × *g*. Then the tumor cells were lysed with ACK lysis buffer (R1010, Solarbio, China) and resuspended in FACS buffer. Finally, after staining with antibodies (PerCP/Cy5.5-conjugated anti-CD45 and APC-conjugated anti-F4/80 antibodies) at 4 °C for 45 min and washing twice with FACS buffer, the cells were resuspended in FACS buffer for FACS analysis. Phagocytizing TAMs (GFP^+^ TAMs) were identified by FACS.

### RNA sequencing of adipose tissues

Six-week-old male C57BL/6 mice were intraperitoneally injected with MC-38 cells (1.0 × 10^6^ cells in 100 µl PBS) and immediately fed a CD or HFD for 5 days. Then the eFats were isolated and used for RNA extraction and sequencing. Each tested sample was pooled from three individual samples. Library construction, sequencing, and data analysis were performed with the help of CapitalBio Technology (Shanghai, China).

### Real-time PCR

Total RNA was extracted (#10296010, Thermo Fisher Scientific) and transcribed into cDNA using PrimeScript (DRR047A, Takara, Dalian, China). Quantitative PCR was performed using a 7900HT Fast Real-Time PCR system. Gene expression was normalized to β-actin expression. Reactions were performed in triplicate using Tli RNaseH plus and universal PCR master mix (#RR820A, TakaRa). Relative expression was calculated with the 2(^–DDCt^) method. The primers can be found in our previous report.^33^

### Statistical analysis

Statistical analyses were carried out using GraphPad Prism 6 (GraphPad Software, Inc.). The survival data were analyzed using Gehan–Breslow–Wilcoxon test. All the other data were expressed as the mean ± s.e.m. and were analyzed using two-tailed unpaired Student’s *t* test or two-way analysis of variance test. For each parameter of all data presented, *P < 0.05, **P < 0.01, and ***P < 0.005.

## Supplementary information

Supplementary information

## Data Availability

All data that support the findings of this study are available from the corresponding author upon reasonable request.
