# Supplementary material for: Dietary fats suppress the peritoneal seeding of colorectal cancer cells through the TLR4/Cxcl10 axis in adipose tissue macrophages
Source: Signal Transduct Target Ther. 2020 Oct 15;5:239. doi: 10.1038/s41392-020-00327-z (PMC7566605; doi:10.1038/s41392-020-00327-z)
Supplement: Supplementary file 1 — Supplementary information [file 41392_2020_327_MOESM1_ESM.docx]

Supplementary Materials for

Dietary fats suppress the peritoneal seeding of colorectal cancer cells through the TLR4/Cxcl10 axis in adipose tissue macrophages

Wei Xiang^1, †^, Rongchen Shi^1, †^, Dapeng Zhang^1^, Xia Kang^1^, Lili Zhang^1^, Jing Yuan^1^, Xuan Zhang^2^, Hongming Miao^1,*^

Correspondence to: hongmingmiao@sina.com

**This PDF file includes:**

Materials and Methods

Figures. S1 to S10

**
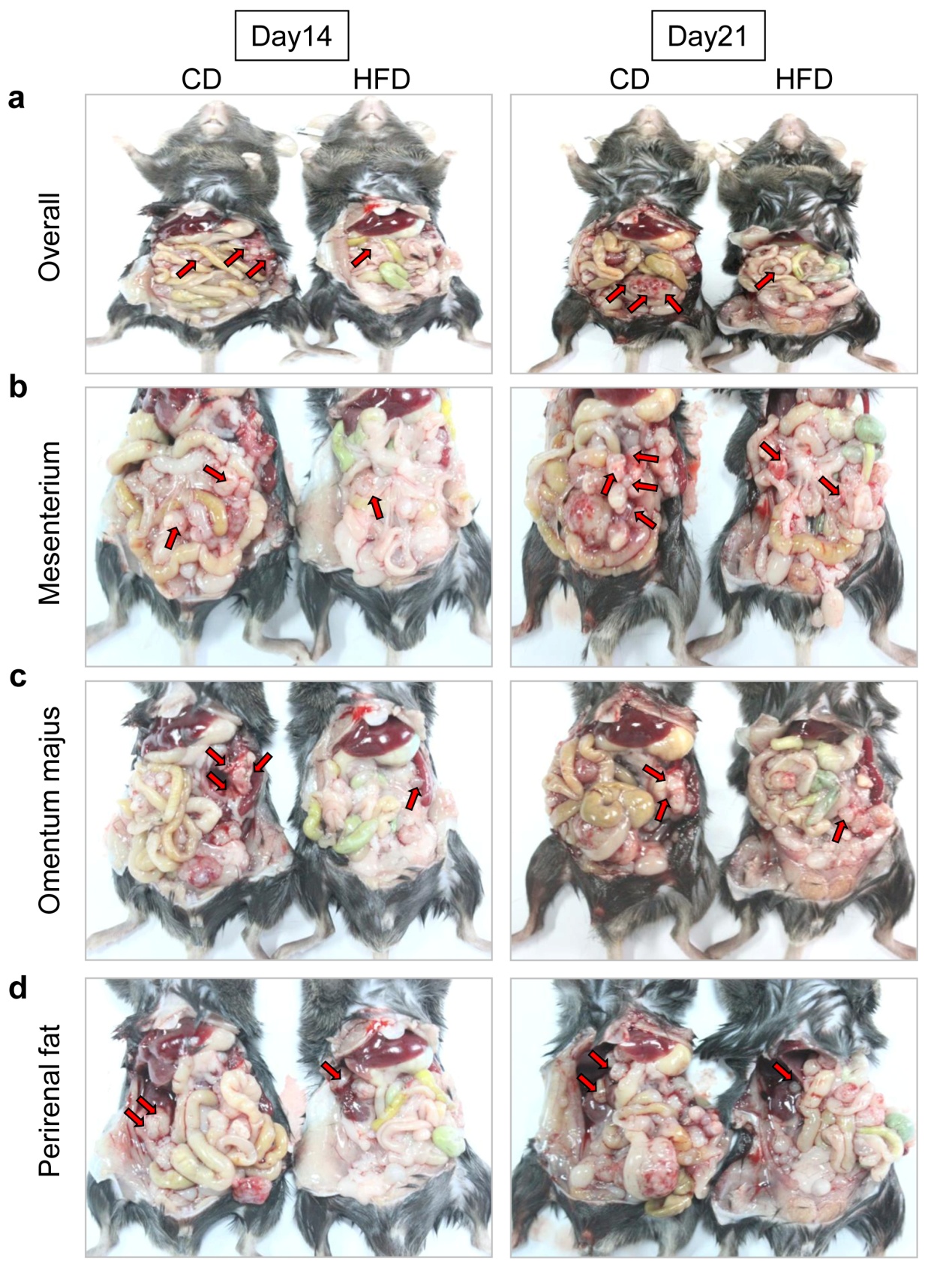
**

Figure. S1.

HFD inhibits the metastatic seeding of colorectal cancer (CRC) cells

(**a-d**) Six-week-old male mice were intraperitoneally inoculated with MC-38 cells (1.0×10^6^ cells in 100 ul PBS for each mouse) and immediately fed with a chow diet (CD) or HFD for 14 or 21 days. Then, the overall status (**a**) as well as tumor nodes in mesenterium (**b**), omentum majus (**c**) and perirenal (**d**) fats were shown. Representative images were displayed. Red arrows indicate the tumor nodes. (n=5)


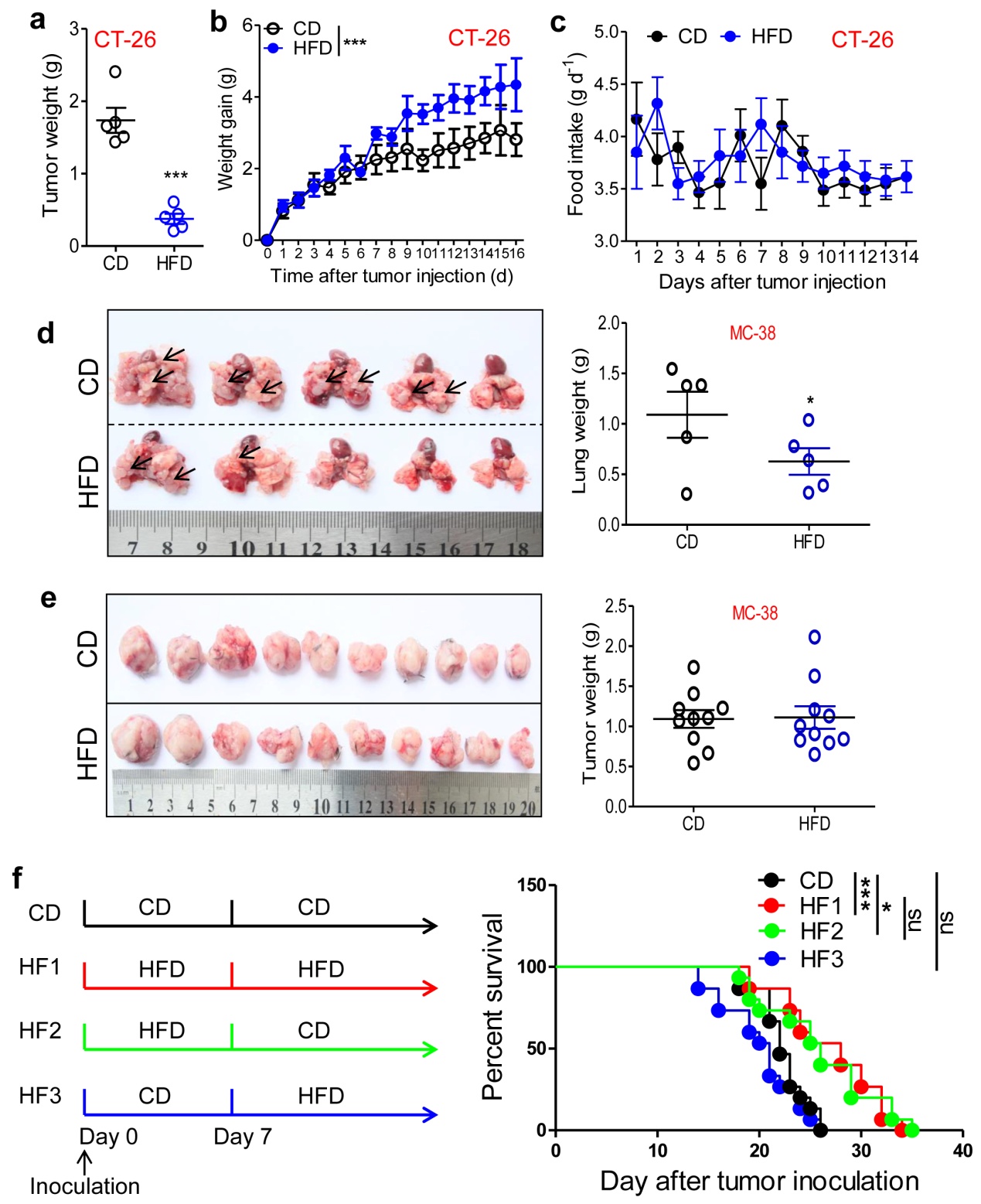


Figure. S2.

HFD inhibits the metastatic seeding of colorectal cancer (CRC) cells

(**a**) HFD inhibits the peritoneal metastasis of CT-26 cells. Six-week-old male mice were intraperitoneally implanted with CT-26 cells (1.0×10^6^ cells in 100 ul PBS) and immediately fed with CD or HFD for 14 days. Then, peritoneal tumor nodes were isolated and calculated in weight. Data showed means±s.e.m. (n=5, ***P<0.005; Student’s t test)

(**b-c**) Weight (**b**) and food intake (**c**) of the mice described in (**a**). Data showed means±s.e.m. (n=5, ***P<0.005; Two-way ANOVA test)

(**d**) HFD inhibits the lung metastasis of CRC cells. Six-week-old male mice were intravenously injected with MC-38 cells (5.0×10^6^ cells in 100 ul PBS) and immediately fed with CD or HFD for 28 days. Then, lungs were isolated and calculated in weight. Arrows indicate the tumor nodes. Data showed means±s.e.m. (n=5, *P<0.05; Student’s t test)

(**e**) Effects of HFD on subcutaneous tumors. Six-week-old male mice were subcutaneously injected with MC-38 cells (5.0×10^6^ cells in 100 ul PBS) and immediately fed with CD or HFD for 14 days. Then, subcutaneous tumors were collected and calculated in weight. Data showed means±s.e.m. (n=10; Student’s t test)

(**f**) HFD improves the survival of tumor-seeded mice in the early phase. After peritoneal engraftment with MC-38 cells, mice were immediately organized into 4 groups with different therapeutic regimens. CD group: mice were treated with chow diet all the way; HF1: mice were treated with high fat diet all the way; HF2: mice were treated with high fat diet for 7 days and then chow diet to the end. HF3: mice were treated with chow diet for 7 days and then high fat diet to the end. Data were means±s.e.m. (n=15, *P<0.05, ***P<0.005; ns, not significant; Gehan-Breslow-Wilcoxon test)


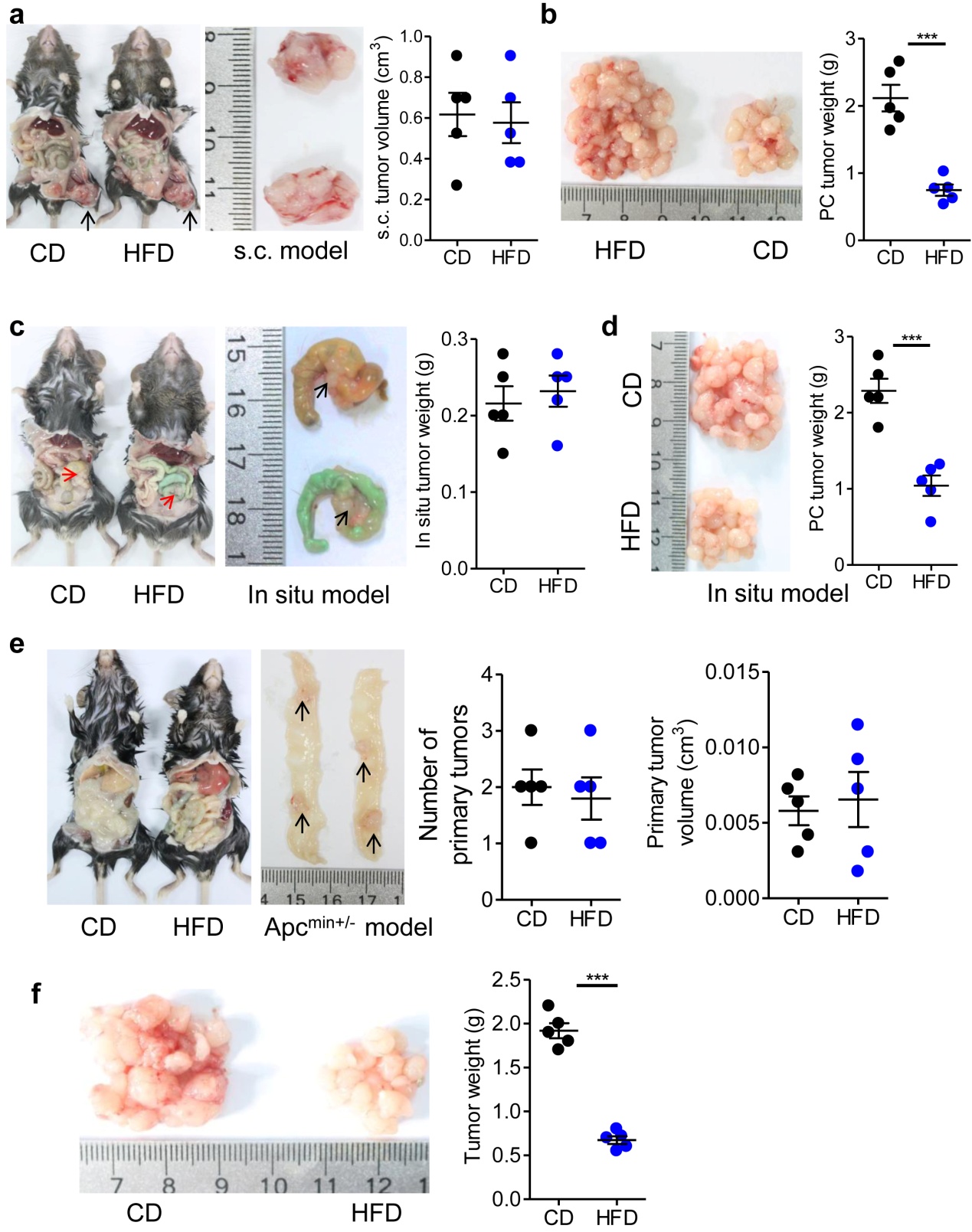


Figure. S3.

HFD inhibits tumor seeding in the models with preexisting primary tumors

(**a-b**) HFD inhibits peritoneal metastasis in the s.c. tumor-seeded model described in Methods. Primary subcutaneous tumors were calculated in volume (**a**) and seeding nodes were measured in weight (**b**). (n=5)

(**c-d**) HFD inhibits peritoneal metastasis in the in situ tumor-seeded model described in Methods. Primary tumors in situ (**c**) and seeding nodes (**d**) were measured in weight. (n=5)

(**e-f**) HFD inhibits peritoneal tumors in the Apc^min+/-^ tumor-seeded model described in Methods. Spontaneous tumors were calculated in number and volume (**e**) and seeding nodes were measured in weight (**f**). (n=5)

Data in (**a-f**) showed means±s.e.m. (***P<0.005, Student’s t test)


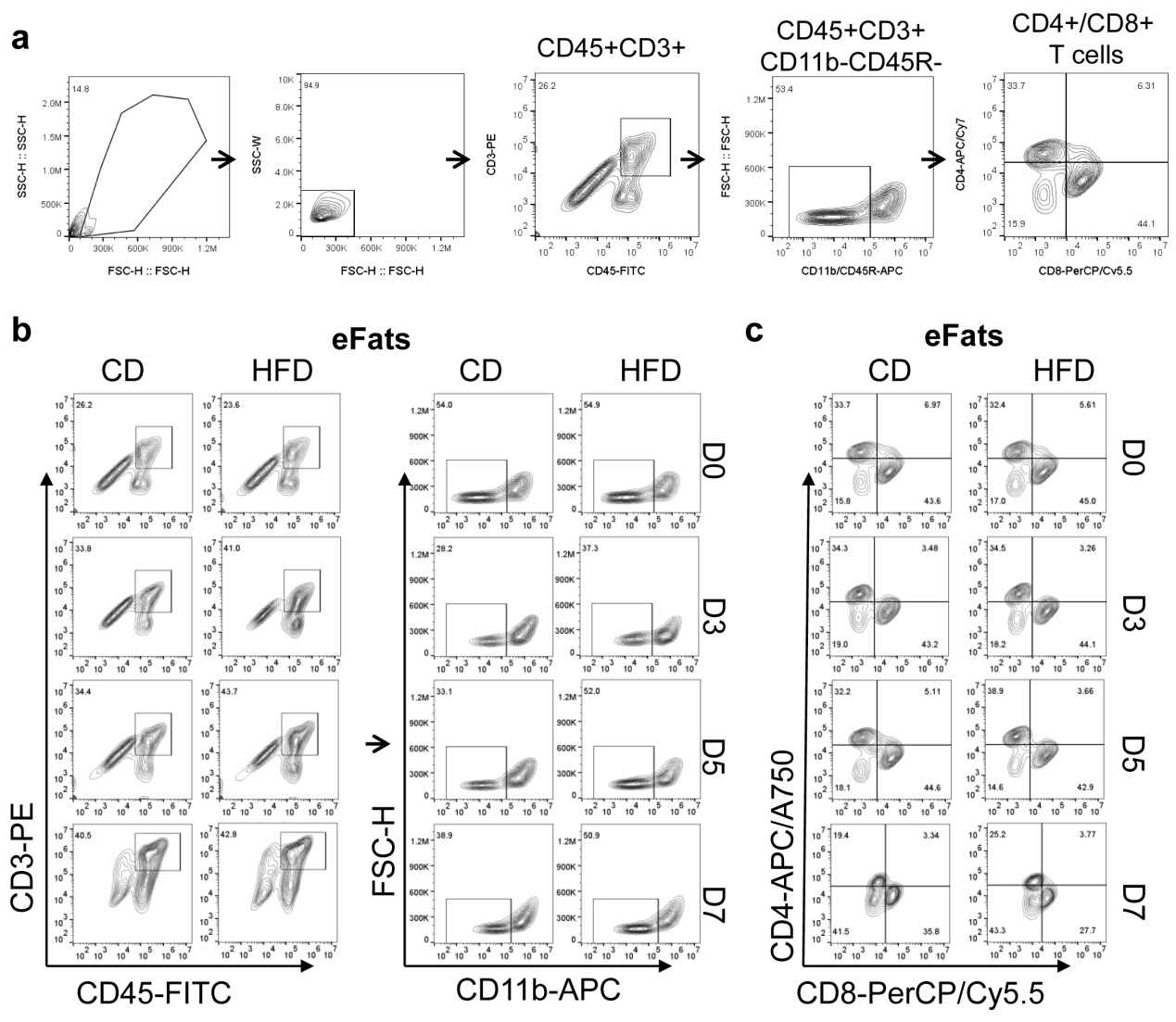


Figure. S4.

Frequencies of T cells in tumor-seeded eFats

(**a**) FACS gating strategy for fat total T cells, CD4^+^ T cells and CD8^+^ T cells in eFats. Debris and doublets were removed, and total T cells were then assessed as CD45^+^CD3^+^CD11b^-^CD45R^-^. CD4^+^ T cells were marked as CD45^+^CD3^+^CD11b^-^CD45R^-^CD4^+^CD8^-^. CD8^+^ T cells were assessed as CD45^+^CD3^+^CD11b^-^CD45R^-^CD4^-^CD8^+^.

(**b-c**) The frequencies of T cells (**b**), CD4^+^ T cells and CD8^+^ T cells (**c**) in tumor-seeded eFats. Six-week-old male mice were intraperitoneally injected with MC-38 cells (1.0×10^6^/100 μl PBS) and immediately fed with CD or HFD. T cells were dynamically counted with flow cytometry as described in (**a**). Representative flow charts were shown.


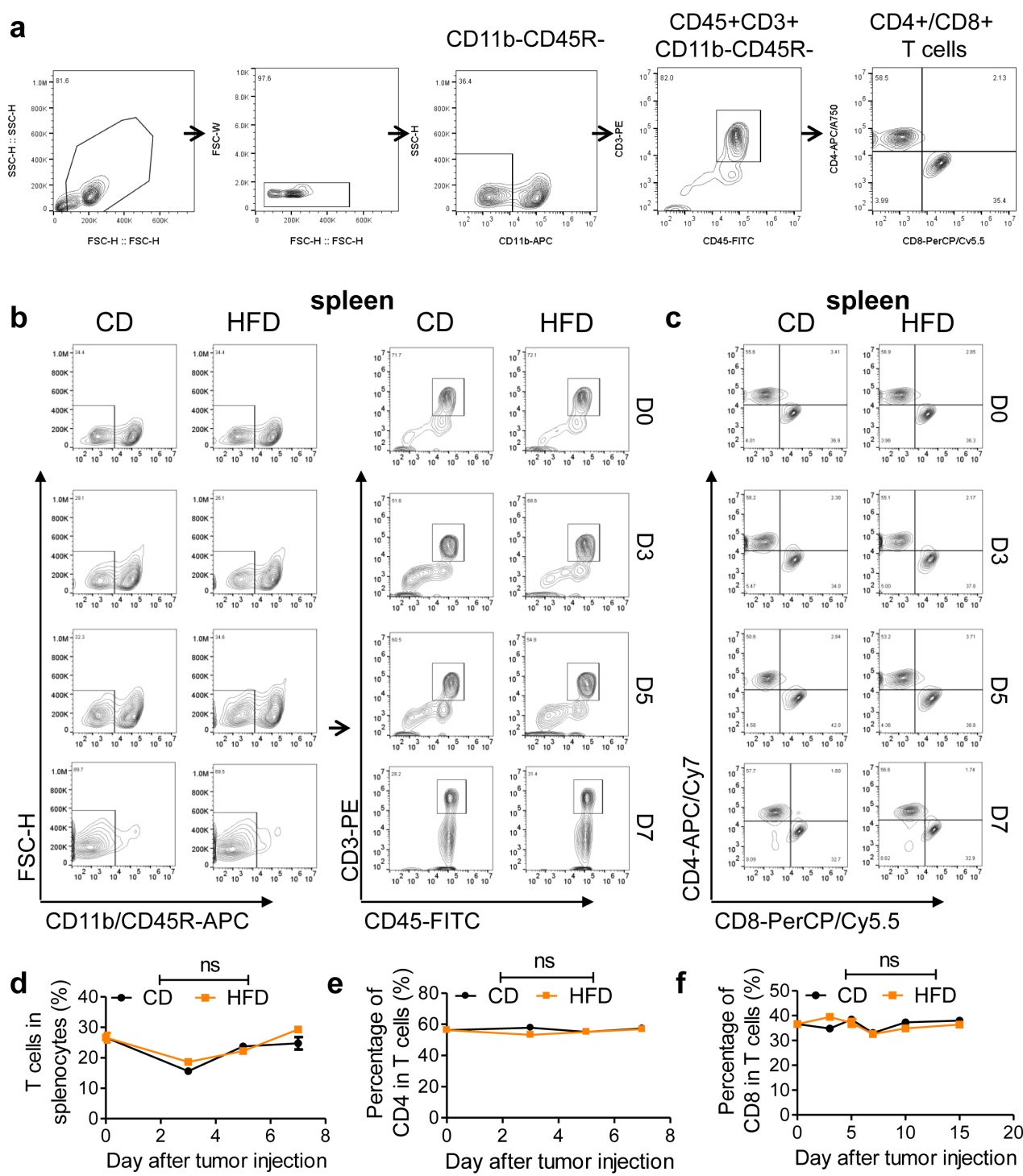


Figure. S5.

Counting of spleen T cells in mice with peritoneal tumor seeding

(**a**) FACS gating strategy for spleen T cells, CD4^+^ T cells and CD8^+^ T cells. Debris and doublets were removed, and spleen T cells were then assessed as CD45^+^CD3^+^CD11b^-^CD45R^-^. CD4^+^ T cells were marked as CD45^+^CD3^+^CD11b^-^CD45R^-^CD4^+^CD8^-^. CD8^+^ T cells were assessed as CD45^+^CD3^+^CD11b^-^CD45R^-^CD4^-^CD8^+^.

(**b-c**) The ratios of spleen T cells (**b**), CD4^+^ T cells and CD8^+^ T cells (**c**) from the CD or HFD-treated mice which were described in supplementary figure 5b-c. Representative flow charts were shown.

(**d-f**) Counting of total T cells (**d**), CD4^+^ T cells (**e**) and CD8^+^ T cells (**f**) in spleens described above in (b-c). Data showed means±s.e.m. (n=3; ns, not significant; Two-way ANOVA test)


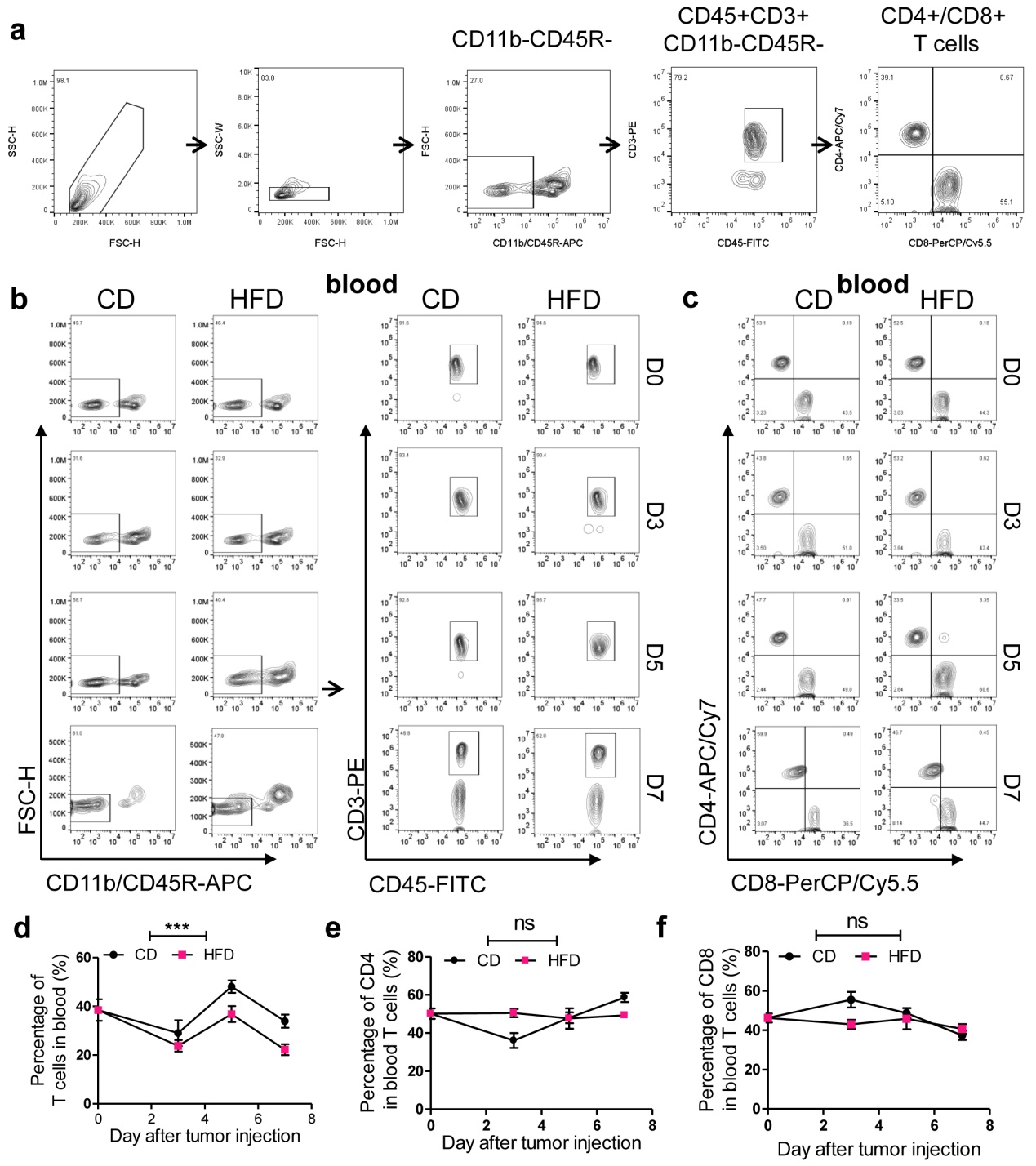


Figure. S6.

Frequencies of blood T cells in peritoneal tumor-seeded mice

(**a**) FACS gating strategy for blood T cells, CD4^+^ T cells and CD8^+^ T cells. Debris and doublets were removed, and blood T cells were then assessed as CD45^+^CD3^+^CD11b^-^CD45R^-^. CD4^+^ T cells were marked as CD45^+^CD3^+^CD11b^-^CD45R^-^CD4^+^CD8^-^. CD8^+^ T cells were assessed as CD45^+^CD3+CD11b^-^CD45R^-^CD4^-^CD8^+^.

(**b-c**) The levels of blood T cells (**b**), CD4^+^ T cells and CD8^+^ T cells (**c**) from the CD or HFD-treated mice which were described in supplementary figure 5b-c. Representative flow charts were shown.

(**d-f**) Counting of total T cells (**d**), CD4^+^ T cells (**e**) and CD8^+^ T cells (**f**) in blood described above in (**b-c**). Data showed means±s.e.m. (n=3, ***P<0.005; ns, not significant; Student’s t test)


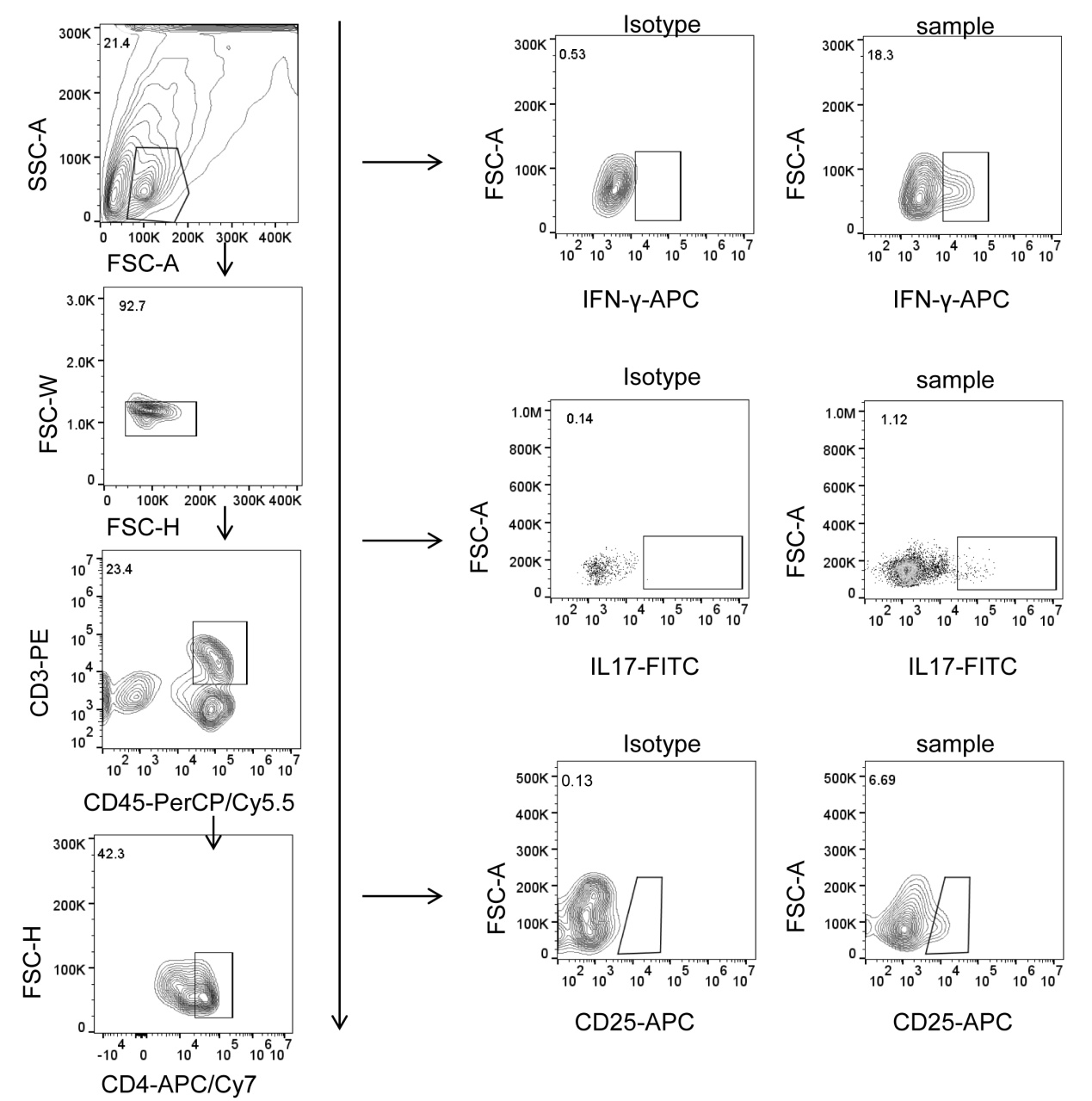


Figure. S7.

Counting of CD4^+^ T cells and the sub-population in mice with tumor seeding

FACS gating strategy for fat T cells, CD4^+^ T cells, IFNγ^+^CD4^+^ T cells, IL-17^+^ CD4^+^ T cells and CD25^+^ CD4^+^ T cells.


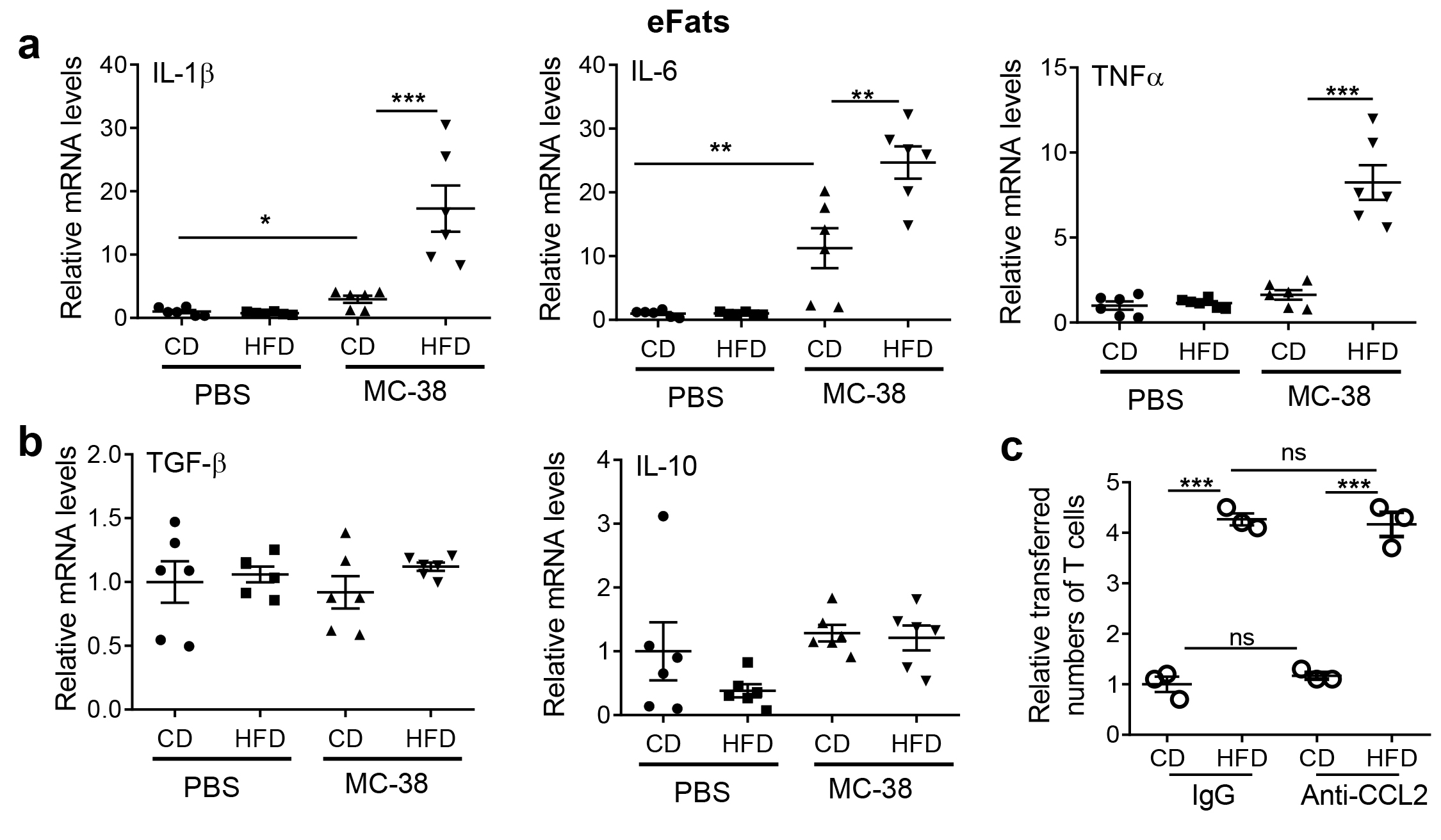


Figure. S8.

HFD induces proinflammatory cytokine expression in visceral fats

(**a**) HFD induces proinflammatory cytokine expression in eFats. Six-week-old male mice were intraperitoneally injected with MC-38 cells (1.0×10^6^ per 100 μl PBS) and immediately fed with CD or HFD for 5 days. mRNA levels of IL-1β, IL-6 and TNFα in eFats were assayed by realtime PCR. (n=6)

(**b**) mRNA levels of anti-inflammatory cytokines TGF-β and IL-10 in the eFats described above in (**a**). (n=6)

(**c**) CCL2 is not required for HFD-induced T cell migration ex vivo. The eFats were isolated from tumor-seeded mice fed a CD or HFD for 5 days. Mouse splenocytes were cultured in the upper chamber, and the eFats were cultured in the lower chamber of a Transwell system. A neutralizing anti-CCL2 antibody (100 ng ml^-1^) was added into the lower chamber to block CCL2 activity. Twenty-four hours later, the migrated T cells in the lower chamber were analyzed by flow cytometry. T cells were defined as CD45^+^CD11b^-^CD3^+^. (n=3)

Data in (**a-c**) showed means±s.e.m. (*P<0.05, **P<0.01 and ***P<0.005; ns, not significant; Student’s t test)


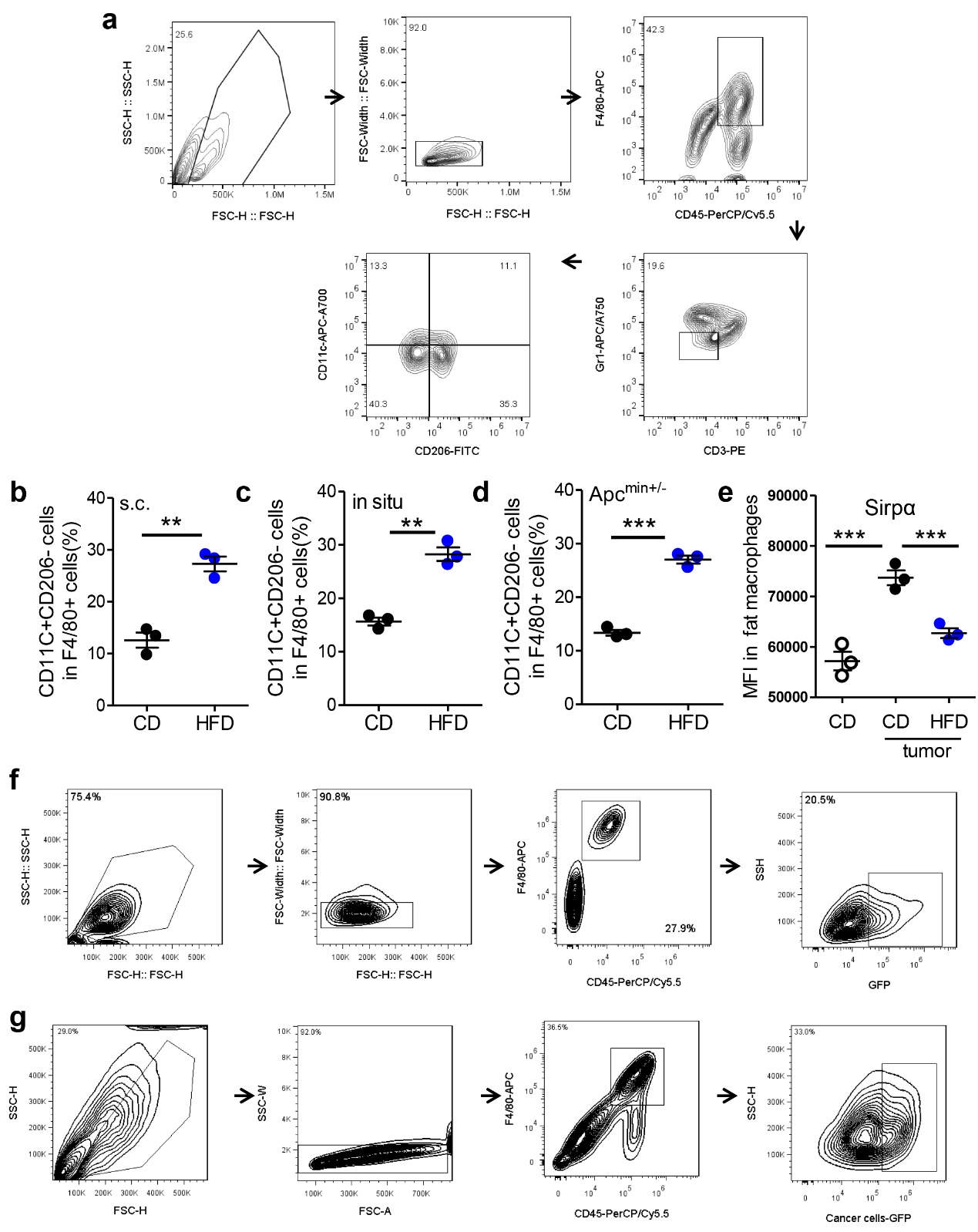


Figure. S9.

Assays of macrophage activity in vitro and in vivo

(**a**) FACS gating strategy for subpopulation of fat macrophages. Debris and doublets were removed, and fat macrophages were then assessed as CD45^+^F4/80^+^CD3^-^Gr1^-^. M1-like macrophages were marked as CD45^+^F4/80^+^CD3^-^Gr1^-^CD11c^+^CD206^-^. M2-like macrophages were assessed as CD45^+^F4/80^+^CD3^-^Gr1^-^CD11c^-^CD206^+^.

(**b-d**) The frequencies of M1-like macrophages in the eFats from the s.c. (**b**), in situ (**c**) or Apc^min+/-^(**d**) tumor-seeded models described in Methods. Each tested sample was pooled from 2 individual ones. (n=3)

(**e**) HFD inhibits the expression of Sirpα in fat macrophages. Six-week-old male mice were intraperitoneally injected with MC-38 cells (1.0×10^6^ per 100 μl PBS) and immediately fed with CD or HFD for 5 days. Then, the expression of macrophage Sirpα in eFats was measured by flow cytometry. (n=3, ***P<0.005)

(**f**) FACS gating strategy for phagocytosis test of macrophages in vitro. Macrophages were marked as CD45^+^F4/80^+^, while MC-38 cells were tagged with GFP.

(**g**) FACS gating strategy for phagocytosis test of tumor associated macrophages in vivo. Macrophages were marked as CD45^+^F4/80^+^, while tumor cells (MC-38) were tagged with GFP.

Data in (**b-e**) showed means±s.e.m. (n=3, **P<0.01 and ***P<0.005; Student’s t test)
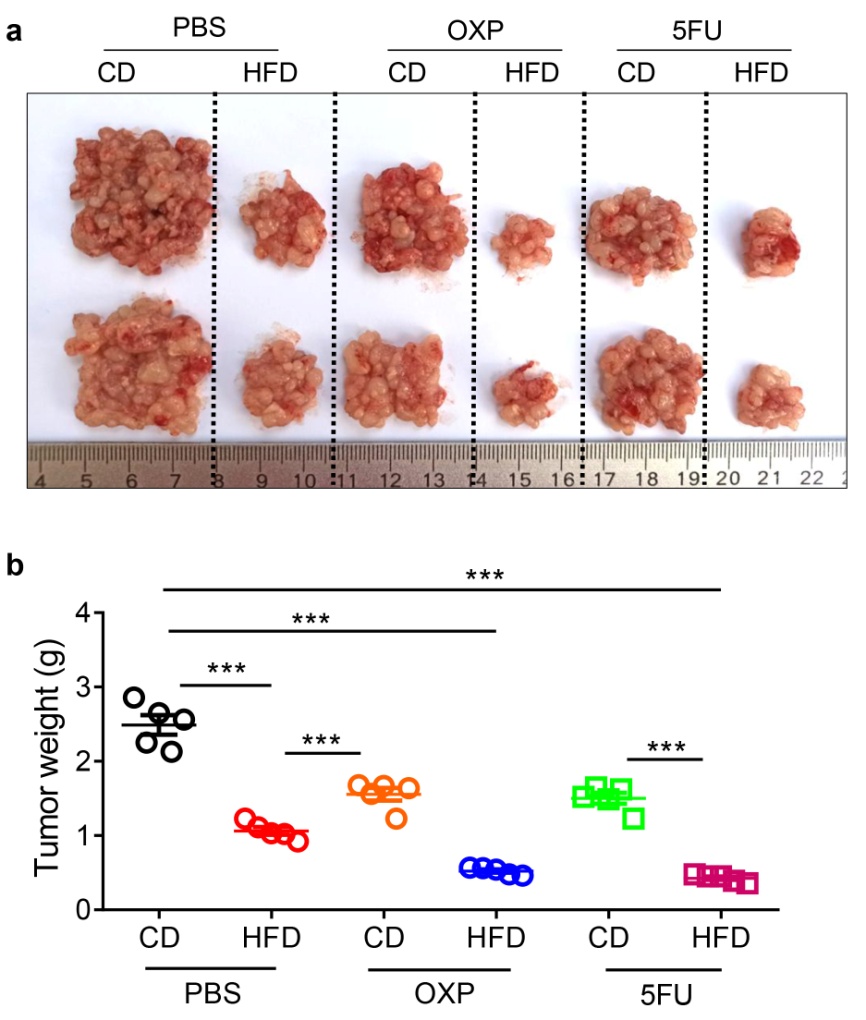


Figure. S10.

HFD and chemotherapeutic drugs synergistically suppress CRC cell seeding in eFats.

(**a-b**) Six-week-old male mice were intraperitoneally inoculated with MC-38 cells (1.0×10^6^ cells in 100 µl PBS per mouse) and immediately fed a CD or HFD (for 7 days) plus oxaliplatin (2.5 mg kg^-1^) or 5-fluorouracil (25 mg kg^-1^) on days 3, 7 and 11. 14 days later, the tumor nodes in the eFats were evaluated. Representative images were displayed (**a**). The tumor weight was calculated (**b**). Data showed means±s.e.m. (n=5, ***P<0.005; Student’s t test)
